# Novel CRK-Cyclin Complex Controls Spindle Assembly Checkpoint in *Toxoplasma* Endodyogeny

**DOI:** 10.1128/mbio.03561-21

**Published:** 2022-02-08

**Authors:** Lauren M. Hawkins, Anatoli V. Naumov, Mrinalini Batra, Changqi Wang, Dale Chaput, Elena S. Suvorova

**Affiliations:** a Division of Infectious Diseases, Department of Internal Medicine, Morsani College of Medicine, University of South Florida, Tampa, Florida, USA; b College of Public Health, University of South Florida, Tampa, Florida, USA; c Proteomics Core, College of Arts and Sciences, University of South Florida, Tampa, Florida, USA; University of Geneva

**Keywords:** Apicomplexa, *Toxoplasma gondii*, endodyogeny, cyclin, cyclin-dependent kinase, mitosis, apicomplexan parasites, cell cycle, protein phosphorylation, spindle assembly checkpoint

## Abstract

Opportunistic parasites of the Apicomplexa phylum use a variety of division modes built on two types of cell cycles that incorporate two distinctive mechanisms of mitosis: uncoupled from and coupled to parasite budding. Parasites have evolved novel factors to regulate such unique replication mechanisms that are poorly understood. Here, we have combined genetics, quantitative fluorescence microscopy, and global proteomics approaches to examine endodyogeny in Toxoplasma gondii dividing by mitosis coupled to cytokinesis. In the current study, we focus on the steps controlled by the recently described atypical Cdk-related kinase T. gondii Crk6 (TgCrk6). While inspecting protein complexes, we found that this previously orphaned TgCrk6 kinase interacts with a parasite-specific atypical cyclin, TgCyc1. We built conditional expression models and examined primary cell cycle defects caused by the lack of TgCrk6 or TgCyc1. Quantitative microscopy assays revealed that tachyzoites deficient in either TgCrk6 or the cyclin partner TgCyc1 exhibit identical mitotic defects, suggesting cooperative action of the complex components. Further examination of the mitotic structures indicated that the TgCrk6/TgCyc1 complex regulates metaphase. This novel finding confirms a functional spindle assembly checkpoint (SAC) in T. gondii. Measuring global changes in protein expression and phosphorylation, we found evidence that canonical activities of the *Toxoplasma* SAC are intertwined with parasite-specific tasks. Analysis of phosphorylation motifs suggests that *Toxoplasma* metaphase is regulated by CDK, mitogen-activated kinase (MAPK), and Aurora kinases, while the TgCrk6/TgCyc1 complex specifically controls the centromere-associated network.

## INTRODUCTION

Apicomplexan parasites are eukaryotic pathogens that cause malaria, toxoplasmosis, and cryptosporidiosis. The active replication of apicomplexans has devastating effects on their human and animal hosts and puts the health of millions of people at risk. Cell division of apicomplexan parasites, such as *Plasmodium* spp., *Cryptosporidium* spp., and Toxoplasma gondii, is strikingly different from the division of their host cells (1–4). The basic landmarks of the eukaryotic cell cycle, such as cell growth (G_1_ phase), DNA replication (S phase), DNA segregation (mitosis), and cytokinesis (budding), are in place. However, the internal structures and regulatory machinery of the apicomplexan cell cycle are novel and specialized. Despite the central role of parasite replication in the pathology of disease, neither the mechanics nor the regulation of the highly specialized apicomplexan cell cycles is understood. To amplify within the human host, the apicomplexan parasites use a sophisticated cell cycle. Apicomplexans violate the basic rule of cell cycle regulation, a “copy once” restriction of DNA replication, and produce from two to thousands of progenies in a single round of division (1–4). Two distinctive mitotic mechanisms determine the scale of the parasite expansion. Multiple rounds of mitosis uncoupled from the budding (cytokinesis) result in genome amplification, while segregation of the chromosomes linked to the bud structures produces infectious daughters (4). Parasites engage both mitotic mechanisms to produce multiple daughters, while endodyogeny, the binary division of the *Toxoplasma* tachyzoites, involves only coupled mitosis and cytokinesis.

To ensure replication fidelity, the apicomplexan parasites, including T. gondii, evolved complex perinuclear structures that provide a physical connection between chromosomes and parasite cytoskeleton (1). The centromeric region of the chromosomes is tightly connected with the centrocone, an intranuclear compartment detected only in Apicomplexan parasites (5). The centrocone is a permanent nuclear membrane structure that expands late in the G_1_ period and splits in mitosis (6, 7). Spindle localization in the centrocone suggests that the split coincides with metaphase-to-anaphase transition in mitosis (8). The *Toxoplasma* centrocone is connected to the bipartite centrosome, comprised of two cores with different protein compositions and distinctive functions (7, 9). The inner core controls nuclear events and the overall centrosome stability, while the outer core bearing centrioles regulates budding. The outer core connection to the conoid via striated fiber provides a physical link to the parasite cytoskeleton (10). How the assembly, duplication, and segregation of these structures are coordinated with cell cycle progression is largely unknown.

The *Toxoplasma* endodyogeny differs from the conventional binary division of model eukaryotes. The conventional cell cycle is unidirectional and regulated by the alternating stage-specific cyclin-dependent kinase (CDK) complexes (11). In contrast, the second half of the T. gondii tachyzoite cycle is braided and is regulated by multiple essential Cdk-related kinases (Crks) (4, 12). Chromosome segregation (mitosis) is concurrent with internal budding (cytokinesis), and both processes are predicted to initiate before chromosome replication is complete in S phase (13, 14). The proper sequence and order of events in mitosis coupled to cytokinesis have never been established due to the absence of a reliable cell cycle synchrony model. Furthermore, the regulation of apicomplexan cell cycle transitions is unknown, because only a few of the major players in cell cycle pathways of conventional eukaryotes are identifiable in apicomplexan genomes (4, 8, 10, 12, 15–21). We have recently discovered multiple Crks that are independently required to complete division of *Toxoplasma* tachyzoites, and most of the apicomplexan Crks are novel factors with limited similarity to known eukaryotic CDKs (12). Mapping *Toxoplasma* Crk activities revealed multiple points of regulation, including parasite-specific mitosis overlapping budding. We discovered a novel Cdk-related kinase, T. gondii Crk6 (TgCrk6), the activity of which is linked to successful progression through mitosis in the tachyzoite cell cycle.

In the current study, we identified atypical cyclin TgCyc1 that forms a complex with TgCrk6. Using robust conditional expression models based on auxin-induced degradation, we determined the role of the atypical mitotic TgCrk6/TgCyc1 complex in *Toxoplasma* endodyogeny. Our findings indicate that TgCrk6 and TgCyc1 coregulate the metaphase-to-anaphase transition in mitosis, which is under the control of the spindle assembly checkpoint (SAC). For the first time, we characterized apicomplexan metaphase coupled to cytokinesis (budding) and identified one of the checkpoint pathways regulated by TgCrk6/TgCyc1.

## RESULTS

### New AID conditional expression model for mitotic TgCrk6 kinase.

**FIG 1:**
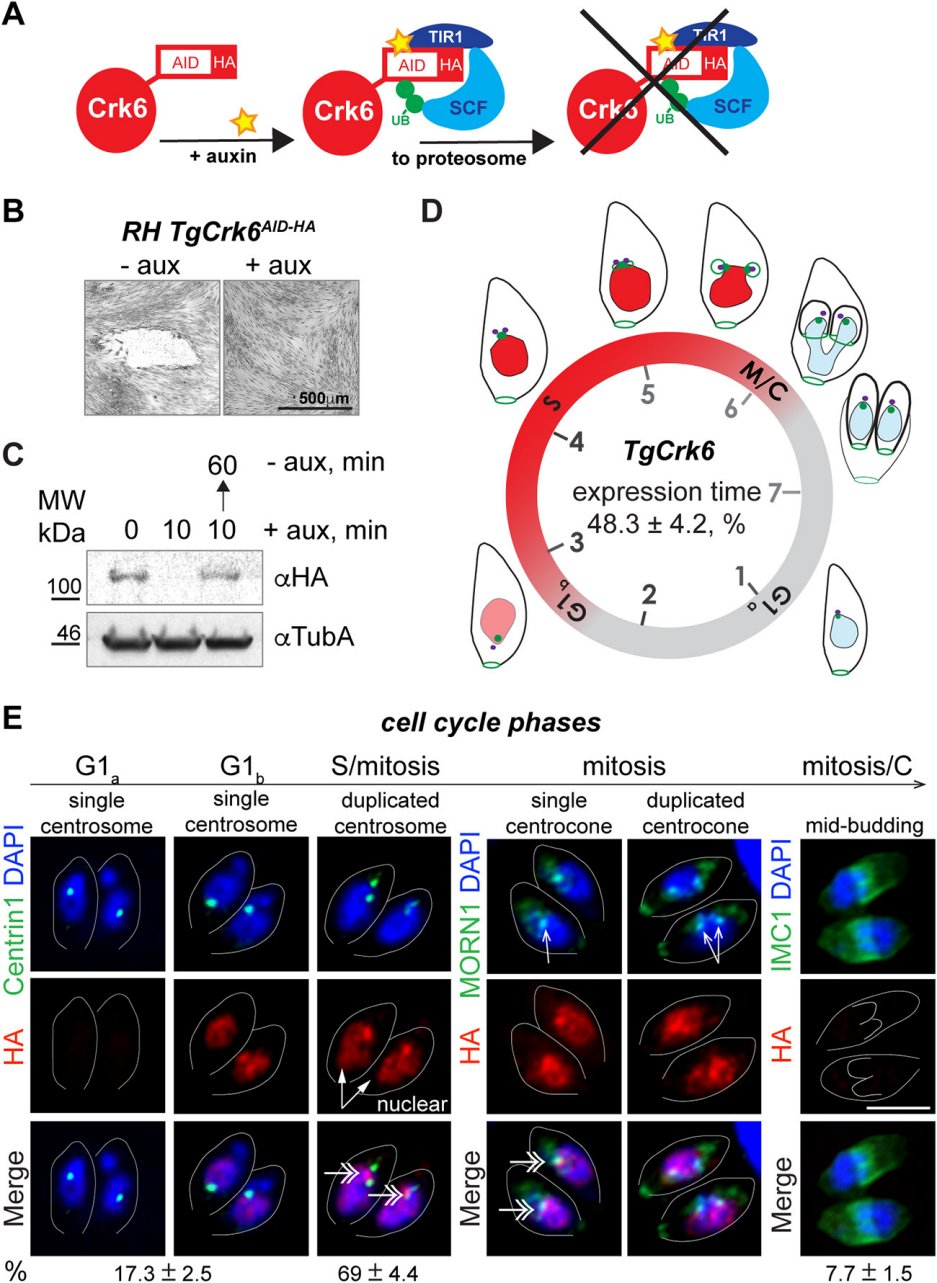
Characterization of the TgCrk6 in the new conditional expression model. (A) Diagram shows the mechanism of auxin-induced degradation of AID-tagged TgCrk6. In the absence of plant hormone auxin (IAA), the native promoter controls TgCrk6^AID-HA^ expression at endogenous levels. Addition of 500 μM auxin (yellow star) promotes interaction of AID-modified TgCrk6 with F-box protein TIR1 (dark blue) and ubiquitin ligase SCF (light blue), resulting in the rapid degradation of TgCrk6^AID-HA^ by proteasome. (B) The differential interference contrast (DIC) images of the HFF monolayers infected with RH TgCrk6^AID-HA^ tachyzoites and grown with or without 500 μM auxin for 7 days. Note that only TgCrk6-expressing tachyzoites (−auxin) formed viable plaques. (C) Western blot analysis of the total lysates of the RHΔ*Ku80TIR1* tachyzoites expressing TgCrk6^AID-HA^. Lysates of nontreated parasites and parasites treated with auxin for 10 min and chased for 1 h were analyzed. Western blots were probed with α-HA (α-rat IgG-HRP) to detect TgCrk6 and with α-tubulin A (α-mouse IgG-HRP) to confirm equal loading of the total lysates. MW, molecular weight. (D) Schematics of the tachyzoite cell cycle. The circle represents 7 h of the tachyzoite division at 37°C and indicates the relative position of the cell cycle phases. Drawings show morphological changes of the dividing tachyzoite. Red color represents the time and primary localization of the TgCrk6 kinase deduced from the quantitative immunofluorescent analysis (see details below). Purple, centrosome (marker, Centrin1); green, centrocone and basal complex (marker, MORN1); blue, nucleus (marker, 4′,6-diamidino-2-phenylindole [DAPI]); black, surface alveoli (marker, IMC1). (E) IF microscopy analysis of TgCrk6^AID-HA^ expression in RHΔ*Ku80TIR1* tachyzoites. To reveal cell cycle-dependent expression, TgCrk6^AID-HA^ (α-HA/α-rat IgG Fluor 568) was costained with centrosomes (α-Centrin1/α-mouse IgG Fluor 488), centrocone (α-MORN1/α-rabbit IgG Flour 488), or alveolar protein IMC1 (α-IMC1/α-rabbit IgG Fluor 488). Cell cycle phases were determined based on the number and morphology of the reference structures, indicated with arrows. Double-headed arrows point to TgCrk6 accumulated in the centrocone. The percentage of the parasites with single or duplicated centrosomes and with internal buds, expressing or not expressing TgCrk6^AID-HA^, was calculated from 3 independent experiments. Mean values ± standard deviations (SD) are shown under corresponding images. Scale bar, 5 μm.

We recently reported that novel TgCrk6 kinase controls the resolution of the spindle compartment, the centrocone, during division of T. gondii tachyzoites (12). Characterization of the cell cycle arrest was done using the tet-OFF model, where downregulation of TgCrk6 was achieved by transcriptional repression over a long period of time, 16 to 24 h (22). Such prolonged deprivation of an essential factor often masks original deficiency. To offset this challenge, we have engineered a new TgCrk6 mutant based on proteolytic control of the factor expression. We chose an auxin-induced degradation model that provides rapid and robust conditional downregulation of the AID (auxin-induced degron)-tagged proteins (Fig. 1A; see also Table S1 in the supplemental material) (23–25). Using a single recombination approach, we modified the endogenous *TgCrk6* genomic locus and introduced an AID motif fused with 3×HA epitopes to the C terminus of the kinase (Fig. S1A and B). Results of the plaque assays validated a new RH TgCrk6^AID-HA^ model and confirmed kinase essentiality. While clonal RH TgCrk6^AID-HA^ transgenic tachyzoites normally grew in the absence of auxin, parasites treated with auxin failed to form plaques, confirming that TgCrk6 is essential for the tachyzoite replication (Fig. 1B). Further analysis of the RH TgCrk6^AID-HA^ tachyzoites confirmed robust TgCrk6^AID-HA^ degradation within 10 min (Fig. 1C). We also demonstrated that the kinase expression can be restored an hour after the auxin removal (Fig. 1C).

10.1128/mbio.03561-21.1 FIG S1 Generation and analysis of the auxin-induced degradation models for TgCrk6 and TgCyc1 in T. gondii RHΔ*Ku80TIR1* strain. (A) Schematics for constructing AID-modified genes. The targeting plasmid included the 3′ fragment of the genomic locus of the gene of interest (GOI) fused with encoded sequence for the miniversion of AID (mAID), 3×HA (HA) epitopes, and the drug selection marker *hxgprt* gene (grey box). Recombination at the target locus is induced by plasmid linearization with a unique endonuclease. Schematics also indicate the relative position of the primers used to conform the GOI’s knock-in in panel B. (B) PCR analysis of the parental RHΔ*Ku80TIR1* and transgenic lines expressing TgCrk6^AID-HA^ or TgCyc1^AID-HA^. The combinations of the primers used to detect either the native or recombined locus are shown. (C) IF microscopy analysis of the TgCrk6^AID-HA^ localization in mitotic RHΔ*Ku80TIR1* tachyzoites. TgCrk6^AID-HA^ (α-HA/α-rat IgG Fluor 568) was costained with centrocone (α-MORN1/α-rabbit IgG Flour 488) and the nuclear dye DAPI (blue). Enlarged images on the right are overlay images in the red and green channel and depict TgCrk6^AID-HA^ expression in the centrocone compartment. Schematics explain the relative location of the analyzed factors. Scale bar, 5 μm. Download FIG S1, TIF file, 1.1 MB. Copyright © 2022 Hawkins et al. 2022 Hawkins et al. https://creativecommons.org/licenses/by/4.0/ This content is distributed under the terms of the Creative Commons Attribution 4.0 International license.

10.1128/mbio.03561-21.5 TABLE S1 Strains, primers, and raw data for figures. Download Table S1, XLSX file, 0.06 MB. Copyright © 2022 Hawkins et al. 2022 Hawkins et al. https://creativecommons.org/licenses/by/4.0/ This content is distributed under the terms of the Creative Commons Attribution 4.0 International license.

Immunofluorescence microscopy analysis showed dominant nuclear localization of the AID-tagged TgCrk6 kinase with temporal accumulation in the centrocone (Fig. 1E and Fig. S1C). However, TgCrk6 was detected only in the subpopulation of the dividing tachyzoites, which indicated cell cycle-dependent oscillation. The quantitative immunofluorescence microscopy analysis (IFA) narrowed the timing of TgCrk6 expression in the second half of the cell cycle (Fig. 1E and Table S1). Costaining of TgCrk6^AID-HA^ with centrosome marker Centrin1 revealed that TgCrk6 was present in nearly 70% of asynchronously dividing tachyzoites with duplicated centrosomes, which marks S-phase and mitotic cells (Fig. 1E, Centrin1) (7). Furthermore, MORN1-positive spindle compartment centrocone matures during S phase and splits in two in mitosis (6, 7). All parasites containing mature or split centrocone were TgCrk6 positive, confirming the kinase expression during S-phase and mitosis (Fig. 1E, MORN1). A small fraction of the TgCrk6-positive parasites contained a single centrosome (17%; Fig. 1E, Centrin1) or had developing buds (8%) (Fig. 1E; IMC1) marking TgCrk6 emergence in late G_1_ phase and degradation in late mitosis coupled to cytokinesis, respectively.

### *Toxoplasma* encodes six families of cyclins.

**FIG 2:**
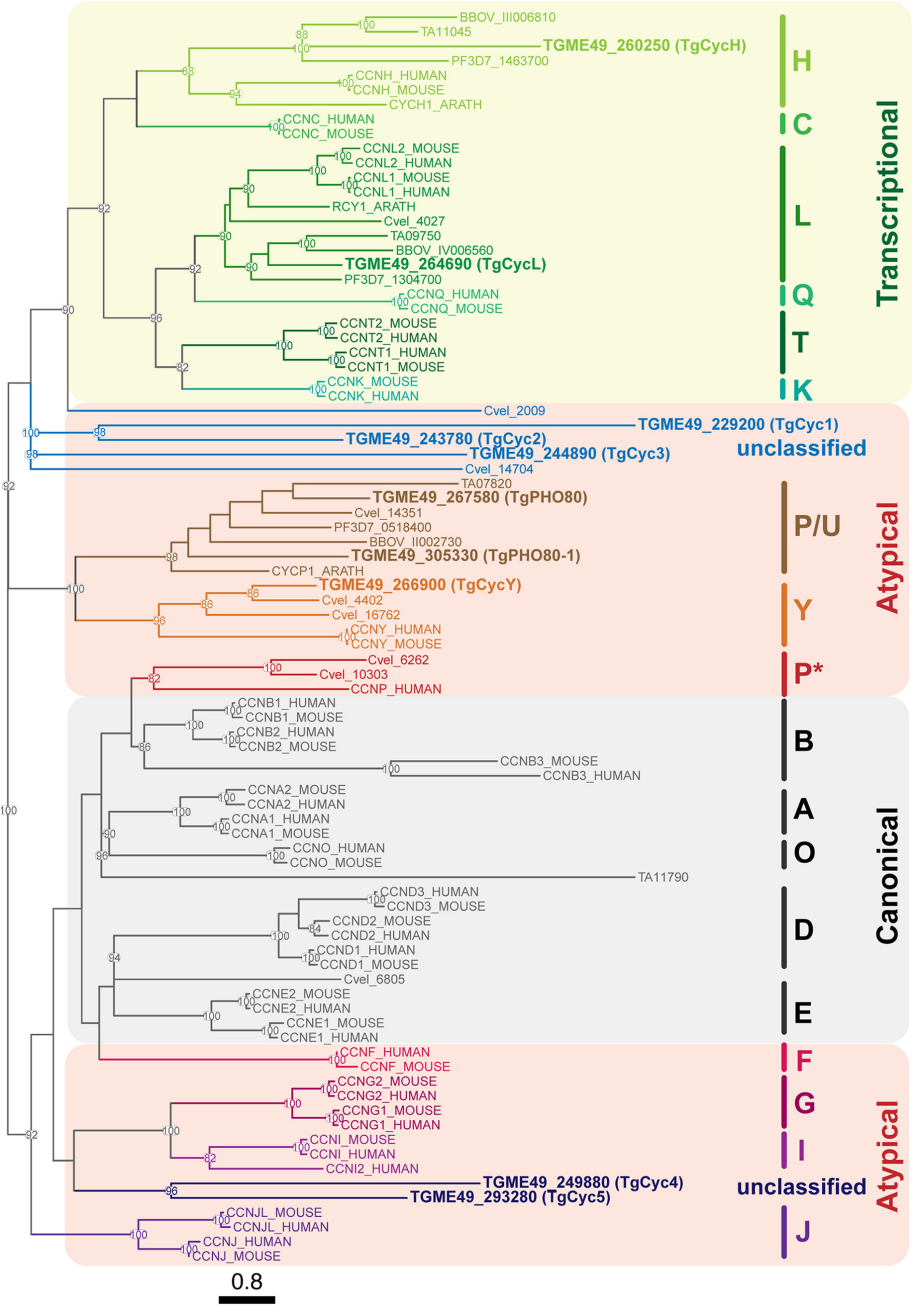
Phylogenetic analysis of apicomplexan cyclins. Phylogenetic tree based on multiple-sequence alignment of predicted cyclins from Toxoplasma gondii, Plasmodium falciparum, Chromera velia, Cryptosporidium parvum, Babesia bovis, Homo sapiens, and Mus musculus. The topology of the phylogenetic tree is optimized by maximum likelihood. Topology support from bootstrapping is shown at nodes. Three classes of cyclins are indicated with different colors. Boldfaced lettering highlights five new T. gondii cyclins.

Our initial analysis of the *Toxoplasma* central cell cycle regulators did not detect a cyclin partner for TgCrk6 (12). However, recent annotations of the T. gondii genome (ToxoDB) revealed several novel cyclin domain-containing proteins, making a total of 10 putative cyclins that we have now included in the new phylogenetic analysis. We compared amino acid sequences of apicomplexan cyclins with the full complement of cyclins of free-living ancestor alveolate (Chromera velia), land plant (Arabidopsis thaliana), and human and mouse cells (Fig. 2 and Table S1). It is worth noting that beyond partial similarity in the cyclin-like domain (∼100 amino acids [aa]), apicomplexan cyclins had no regions in common with other eukaryotic cyclins. Our results confirmed that apicomplexan parasites lack canonical A, B, D, and E cyclins, known for their role in cell cycle regulation (26, 27). The only preserved cyclin families include transcriptional regulators (H and L cyclins) and atypical cyclins related to P/U and Y families. In our analysis, five *Toxoplasma* cyclins, including newly identified members, did not show a relationship with any known family and were clustered with atypical cyclins. Therefore, we decided to name these unclassified cyclin domain-containing proteins TgCyc1 through TgCyc5 (Fig. 2). Five atypical *Toxoplasma* cyclins formed two unrelated groups. TgCyc1 appeared to be related to TgCyc2, and, along with TgCyc3, may have had an ancestor in common with transcriptional cyclins. TgCyc4 and TgCyc5 seem to be related and may have derived from the same ancestor as the group of G/I cyclins. Our phylogenic analysis suggests early emergence of atypical cyclins in apicomplexans that further evolved to accommodate specific needs of individual parasite families.

### TgCrk6 forms complex with novel cyclin.

**FIG 3:**
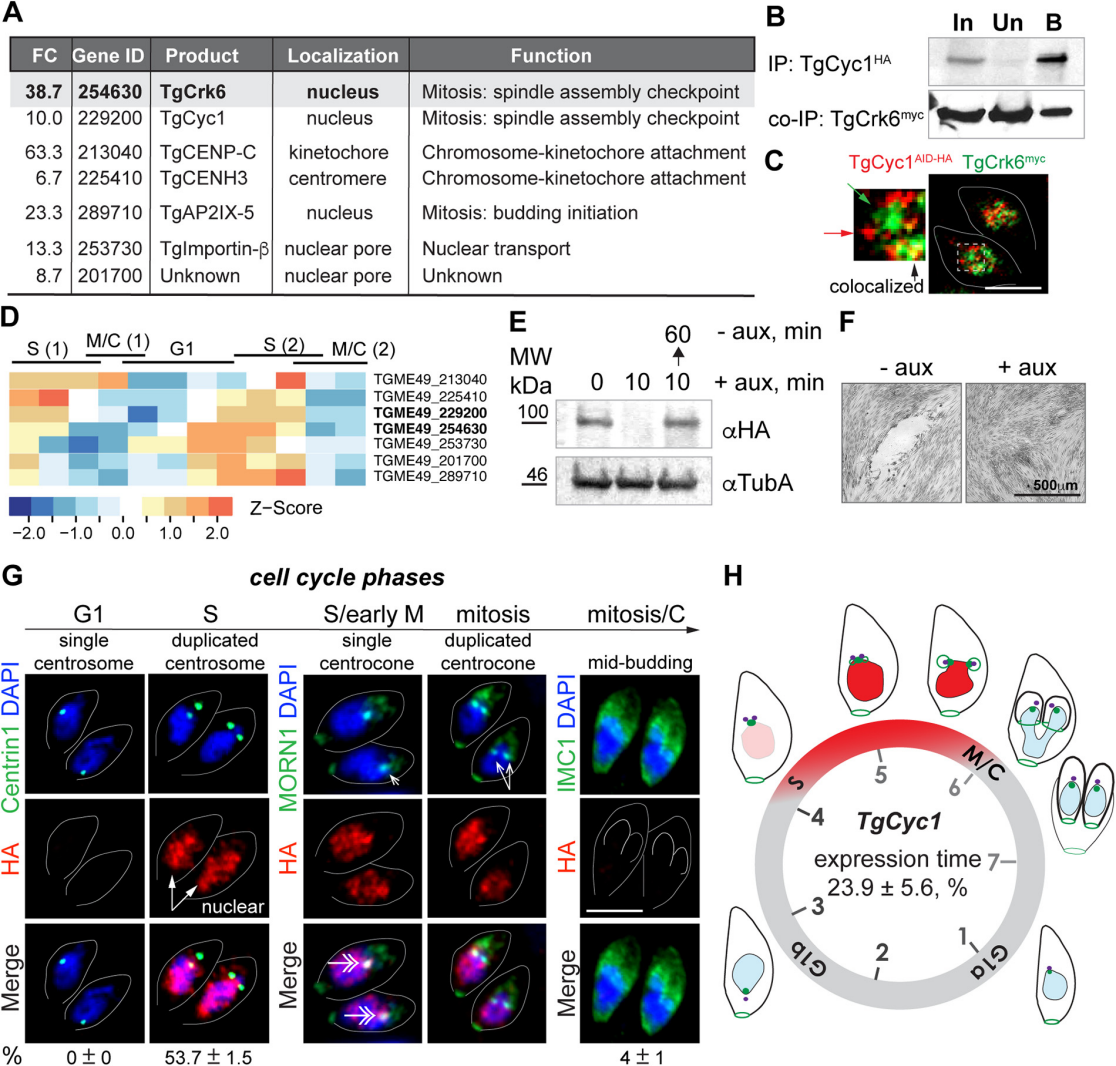
TgCrk6 interacts with novel mitotic cyclin TgCyc1. (A) Selected TgCrk6 interactors identified by mass spectroscopy analysis of isolated TgCrk6 complexes. The table depicts selected hits predicted to localize to the nucleus. FC, fold change of the peptides in the TgCrk6 complexes. IP, immunoprecipitation. (B) TgCyc1 forms a complex with TgCrk6. TgCyc1/TgCrk6 complexes were immunoisolated from the soluble fraction (In; input) of parasites coexpressing endogenous TgCyc1^AID-HA^ and TgCrk6^myc^. Beads with precipitated complexes (B) and depleted soluble fraction (Un) (unbound) were probed with α-myc (α-rabbit IgG-HRP) and α-HA (α-rat IgG-HRP) antibodies to detect interacting TgCrk6 and to confirm efficient pulldown of TgCyc1 (the IP panels at the top). (C) IFA images of tachyzoites coexpressing endogenous TgCyc1^AID-HA^ and TgCrk6^myc^. Parasites were costained with α-HA/α-rat IgG Fluor 568 and α-myc/α-rabbit IgG Fluor 488. The enlarged overlay image shows an example of colocalization (black arrow) and separate localization, indicated with the corresponding color of the arrows. Scale bar, 5 μm. (D) Selected nuclear TgCrk6 interactors from panel A are maximally expressed in S phase and mitosis. Heatmap shows transcriptional signatures of selected genes across tachyzoite cycle of T. gondii. The transcriptional profile of each gene is normalized by *z* score. (E) Western blot analysis of the total lysates of the RHΔ*Ku80TIR1* tachyzoites expressing TgCyc1^AID-HA^. Lysates of nontreated parasites and parasites treated with auxin for 10 min and chased for 1 h were analyzed. Western blots were probed with α-HA (α-rat IgG-HRP) to detect TgCyc1 and with α-tubulin A (α-mouse IgG-HRP) to confirm equal loading of the total lysates. (F) The new cyclin essentiality was tested by the ability of RH TgCyc1^AID-HA^ mutant parasites to form plaques after 7 days with or without 500 μM auxin. The representative DIC images are shown. Note that only TgCyc1-expressing tachyzoites (−auxin) formed viable plaques. (G) IF microscopy analysis of TgCyc1^AID-HA^ expression in RHΔ*Ku80TIR1* tachyzoites. To establish the time frame for TgCyc1 expression, TgCyc1^AID-HA^ (α-HA/α-rat IgG Fluor 568) was costained with centrosomes (α-Centrin1/α-mouse IgG Fluor 488), centrocone (α-MORN1/α-rabbit IgG Flour 488), or alveolar protein IMC1 (α-IMC1/α-rabbit IgG Fluor 488). Cell cycle phases were determined based on the number and morphology of the reference structures, indicated with arrows. Double-headed arrows point to TgCyc1 accumulated in the centrocone. The percentage of the parasites with a single or duplicated centrosome and with internal buds, expressing or not expressing TgCyc1^AID-HA^, was calculated from 3 independent experiments. Mean value ±SD is shown under corresponding images. Scale bar, 5 μm. (H) Schematics of the tachyzoite cell cycle. The circle represents 7 h of the tachyzoite division at 37°C and indicates the relative position of the cell cycle phases. Drawings show morphological changes of the dividing tachyzoite. Red color represents the time and primary localization of the novel cyclin TgCyc1, deduced from the quantitative immunofluorescent analysis (see details for panel G). Purple, centrosome (marker, Centrin1); green, centrocone and basal complex (marker, MORN1); blue, nucleus (marker, DAPI); black, surface alveoli (marker, IMC1).

To determine whether new putative *Toxoplasma* cyclins interact with TgCrk6, we performed mass spectrometry analysis of the TgCrk6^AID-HA^ complexes isolated from dividing tachyzoites (Fig. S2A). Out of 80 proteins specifically associated with TgCrk6, proteins with unknown function constituted the larger portion of the TgCrk6 interactome (Table S2). The component enrichment analysis of localization showed that TgCrk6 predominantly interacts with nuclear factors, many of which, similar to TgCrk6, are predicted to peak in the second half of the cell cycle (Fig. 3D and Fig. S3B and C) (28). We previously showed and now confirmed that TgCrk6 temporally accumulates in the centrocone region strategically important for mitosis (Fig. S1C) (12). Centrocone is aligned with bundled centromeres (5, 7, 18). Among prominent TgCrk6 interactors, we detected proteins localized in the centromere, TgCenH3, and the factor analogous to inner kinetochore protein CENP-C in conventional eukaryotes (Fig. 3A) that may explain TgCrk6 accumulation in the centrocone. The structure and function of *Toxoplasma* centrocone remain poorly understood. Nevertheless, it has been shown that a specialized nuclear pore is located near the centrocone (19). In good agreement with this finding, we detected two nuclear pore proteins (TGME49_253730 and TGME49_201700) in the TgCrk6^HA^ complexes. We also detected TgCrk6 interaction with DNA-binding factors of the AP2 family that coincide with TgCrk6 accumulation in the large nuclear complexes due to association with chromatin (Fig. S1C).

10.1128/mbio.03561-21.2 FIG S2 Analysis of the TgCrk6 interactome. (A) Immunoblot image shows a typical result of the TgCrk6^AID-HA^ immunoprecipitation. The kinase complexes were isolated from the soluble fraction (In) of parasites expressing endogenously tagged TgCrk6^AID-HA^. The input fraction (In), beads with precipitated complexes (B), and depleted soluble fraction (Un) (unbound) were probed with α-HA (α-rat IgG-HRP) antibodies to confirm efficient pulldown. The asterisk shows products of the TgCrk6^AID-HA^ degradation. Isolated TgCrk6 protein complexes were examined by mass spectrometry. (B) Pie chart of the predicted localization of the putative TgCrk6 interactors. (C) Heatmap shows cell cycle expression profiles of the putative TgCrk6 interactors. Download FIG S2, TIF file, 2.6 MB. Copyright © 2022 Hawkins et al. 2022 Hawkins et al. https://creativecommons.org/licenses/by/4.0/ This content is distributed under the terms of the Creative Commons Attribution 4.0 International license.

10.1128/mbio.03561-21.3 FIG S3 Mitotic arrest caused by the TgCrk6 and TgCyc1 deficiency. (A) IFA images of the RH TgCrk6^AID-HA^ tachyzoites not treated (no auxin) or treated with 500 μM auxin for 7 h. To examine centrosome duplication, DNA segregation, and overall cell morphology, parasites were costained with α-IMC1/α-rabbit IgG Fluor 488, DAPI (blue), and α-Centrin1/α-mouse IgG Fluor 568 antibodies and stains. The arrows point to indicated abnormalities. Mitotic phases were identified based on the intensity of the DNA staining, centrosome number and arrangement, and the presence of internal buds. Scale bar, 5 μm. (B) IFA images of the RH TgCrk6^AID-HA^ and RH TgCyc1^AID-HA^ tachyzoites not treated (no auxin) or treated with 500 μM auxin for 4 h. Scale bar, 5 μm. Download FIG S3, TIF file, 2.8 MB. Copyright © 2022 Hawkins et al. 2022 Hawkins et al. https://creativecommons.org/licenses/by/4.0/ This content is distributed under the terms of the Creative Commons Attribution 4.0 International license.

10.1128/mbio.03561-21.6 TABLE S2 Analysis of the TgCrk6 protein complexes. Download Table S2, XLSX file, 0.6 MB. Copyright © 2022 Hawkins et al. 2022 Hawkins et al. https://creativecommons.org/licenses/by/4.0/ This content is distributed under the terms of the Creative Commons Attribution 4.0 International license.

The isolated TgCrk6^HA^ complexes contained a single cyclin-like protein, TgCyc1 (TGME49_229200). To confirm TgCrk6-TgCyc1 interaction, we established a transgenic line coexpressing endogenously tagged TgCyc1^AID-HA^ and TgCrk6^myc^ (Fig. S1B, Table S1). Protein complexes formed by cyclin TgCyc1^AID-HA^ were immunoisolated and probed for the presence of TgCrk6^myc^ kinase. Western blot analysis verified reciprocal TgCyc1-TgCrk6 interaction, and the partnership was further confirmed by immunofluorescence microscopy (Fig. 3B and C). Similar to TgCrk6^myc^, TgCyc1^HA^ was predominantly expressed in the nucleus, where it partially overlapped with interacting TgCrk6 kinase (Fig. 3C). TgCyc1 was also transiently accumulated in the centrocone region (Fig. 3G, costaining with MORN1). Altogether, the colocalization, mass spectrometry, and Western blot analyses confirmed that TgCrk6 and TgCyc1 form a complex in dividing tachyzoites.

### Tachyzoite replication requires mitotic cyclin TgCyc1.

To characterize the novel atypical cyclin, we created an AID model of the conditional TgCyc1 expression (Fig. S1B, Table S1). Western blot analysis showed that, comparable to AID-tagged TgCrk6 kinase, cyclin TgCyc1^AID-HA^ rapidly degrades within 10 min of auxin addition, and cyclin expression was restored during 1 h of auxin removal (Fig. 3E). Plaque assay of the RH TgCyc1^AID-HA^ parasites grown in the absence or presence of auxin for 7 days revealed that TgCyc1 is essential for tachyzoite survival. Auxin-treated, TgCyc1-deficient parasites were unable to propagate and, consequently, did not form visible plaques (Fig. 3F). Like TgCrk6, cyclin TgCyc1 was maximally expressed during mitosis but in a much narrower window than that of its interacting TgCrk6 kinase (Fig. 3G and H). While TgCrk6 remained in tachyzoites during half of its division cycle (48%), the interacting cyclin TgCyc1 was only detected during 24% of replication time (Fig. 3H and Table S1). Quantitative IFA using cell cycle markers Centrin1 (centrosome), MORN1 (centrocone/basal body), and IMC1 (surface alveoli) refined the temporal TgCyc1 expression (6, 7, 29). We established that TgCyc1 emerged late in S phase (cells with duplicated centrosomes) and was entirely degraded by early budding (cells with small internal buds) (Fig. 3G). TgCyc1 reached a peak of expression at the time of the centrocone split (prior two MORN1-positive intranuclear dots become visible), which marks the metaphase-to-anaphase transition in *Toxoplasma* mitosis (Fig. 3G) (6, 7, 12). Our findings demonstrated the dynamic expression of the novel atypical TgCyc1 cyclin, the localization and expression of which coincide with interacting TgCrk6 kinase.

### The lack of TgCyc1 results in mitotic deficiency.

**FIG 4:**
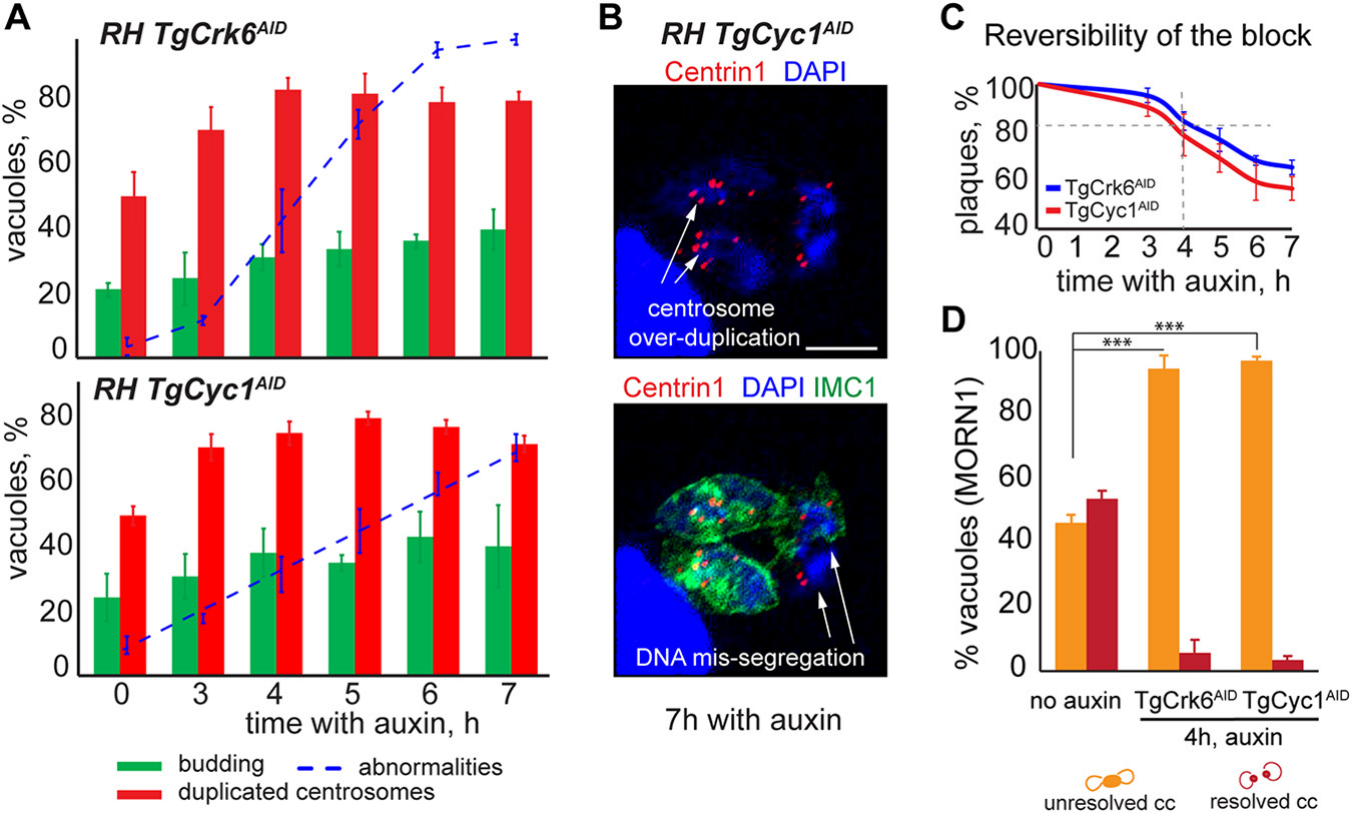
Lack of TgCrk6 or TgCyc1 leads to similar arrest in mitosis. (A) The RH TgCrk6^AID-HA^ (upper graph) or RH TgCyc1^AID-HA^ (lower graph) tachyzoites were examined by IFA after 3, 4, 5, 6, and 7 h with 500 μM auxin and compared to the untreated population (0 h). Centrosome duplication and budding were evaluated using α-Centrin1 (red bars) and α-IMC1 (green bars) antibody. Parasites were costained with DAPI to detect nuclear morphology. All markers were accounted for to quantify abnormalities of the cell division (blue dotted line): DNA missegregation and centrosome overduplication. Each cell cycle marker was examined in 100 random vacuoles in three independent experiments. The mean and SD values are show on the plots. (B) The IFA images depict cellular abnormalities accumulated after 7 h of TgCyc1 depletion (7 h, auxin). Parasite, nuclear morphology, and centrosome numbers were detected with α-IMC1/α-rabbit IgG fluor 488, DAPI (blue), and α-Centrin1/α-mouse IgG Fluor 568 antibodies and stains. Scale bar, 5 μm. (C) The reversibility of the mitotic block induced by TgCrk6 or TgCyc1 withdrawal was determined by plaque assay. Freshly invaded RH TgCrk6^AID-HA^ (blue line) or RH TgCyc1^AID-HA^ (red line) parasites were incubated with 500 μM auxin for the indicated times before the medium was replaced with normal growth medium without auxin to allow for plaque development. Plaque numbers represent averages from three independent measurements. (D) Quantification of the primary mitotic defect caused by RH TgCrk6^AID-HA^ or RH TgCyc1^AID-HA^ deficiency. Unresolved (orange bars) and resolved (red bars) centrocones were quantified based on the MORN1 and IMC1 costaining shown in panel B. One hundred random vacuoles of the parasites containing a single centrocone dot with two attached rings (metaphase) or two centrocone dots with an attached ring (anaphase and early budding) were examined in three independent experiments. The mean values are plotted on the graph.

Our previous work indicated that kinase TgCrk6 regulates mitotic progression in *Toxoplasma* tachyzoites (12). To find out whether TgCrk6 and TgCyc1 coregulate mitosis, we compared cell cycle deficiencies caused by the lack of TgCrk6 or TgCyc1. We took advantage of the rapid protein degradation offered by an auxin-induced degradation approach where TgCrk6^AID-HA^ and TgCyc1^AID-HA^ were eliminated within 10 min of auxin treatment and set to monitor the development of the cell cycle block for one division cycle (7 h). The time course examination showed that similar to TgCrk6, downregulation of TgCyc1 resulted in the post-S-phase block (Fig. 4A). After 4 h of either factor deficiency, over 80% of tachyzoites had duplicated centrosomes, a hallmark of the S-phase, mitosis, and early budding stages (Fig. 4A, red bars on the lower graph) (7). Since mitosis in tachyzoites is coupled to cytokinesis, we also examined how the lack of TgCrk6 and TgCyc1 affected budding. We found that neither TgCrk6 nor TgCyc1 deficiency significantly affected concurrent budding processes. Over 7 h of auxin treatment, the population of the vacuoles undergoing budding steadily grew by 20%, suggesting that TgCrk6-TgCyc1 is unlikely to control cytokinesis (Fig. 4A and B and Table S1).

The TgCrk6 or TgCyc1 depletion time directly correlated with the number of vacuoles displaying morphological abnormalities, such as overamplification of the centrosomes, indicative of the cells reentering mitosis and chromosome missegregation, leading to the loss of DNA in the extracellular space (Fig. 4B and Fig. S3A). Significant accumulation of the defects (over 30% vacuoles) occurred after 4 h of either factor elimination, at which time parasites approached a reliable cell cycle arrest. Quantitative analysis showed no significant change in centrosome duplication or budding events past 4 h of incubation with auxin. Thus, detected abnormalities were likely the secondary effects of the TgCrk6 or TgCyc1 depletion and may mask the primary phenotype. In agreement with this observation, we determined that parasites had a high rate of survival of the block induced by 4 h of auxin treatment. Over 80% of vacuoles lacking either factor returned to normal growth efficiency (Fig. 4C and Table S1). Therefore, in further studies we focused on analysis of the 4-h TgCrk6 and TgCyc1 deficiency that results in enrichment of mitotic parasites with minimum secondary effect.

### TgCrk6 and TgCyc1 control metaphase-to-anaphase transition in tachyzoites.

Analyzing the TgCrk6 tet-OFF model, we previously found that TgCrk6 is required for efficient resolution of the spindle compartment centrocone (12). To find out whether the loss of the interacting cyclin TgCyc1 results in a similar mitotic defect, we compared the state of the centrocone in the auxin-arrested TgCyc1^AID-HA^ and TgCrk6^AID-HA^ parasites. Costaining of the factor-deficient tachyzoites with centrocone marker MORN1 and surface alveoli marker IMC1 revealed that TgCrk6- and TgCyc1-depleted parasites indeed have similar deficiency of the centrocone resolution in late budding (Fig. S3B). This finding strongly supports the cooperative action of novel kinase TgCrk6 and atypical cyclin TgCyc1 in regulation of the tachyzoite mitosis. Further quantitative analysis of the centrocone morphology showed that over 90% of TgCrk6- or TgCyc1-deficient tachyzoites contained a single centrocone, suggesting a robust metaphase block (Fig. 4D and Table S1).

**FIG 5:**
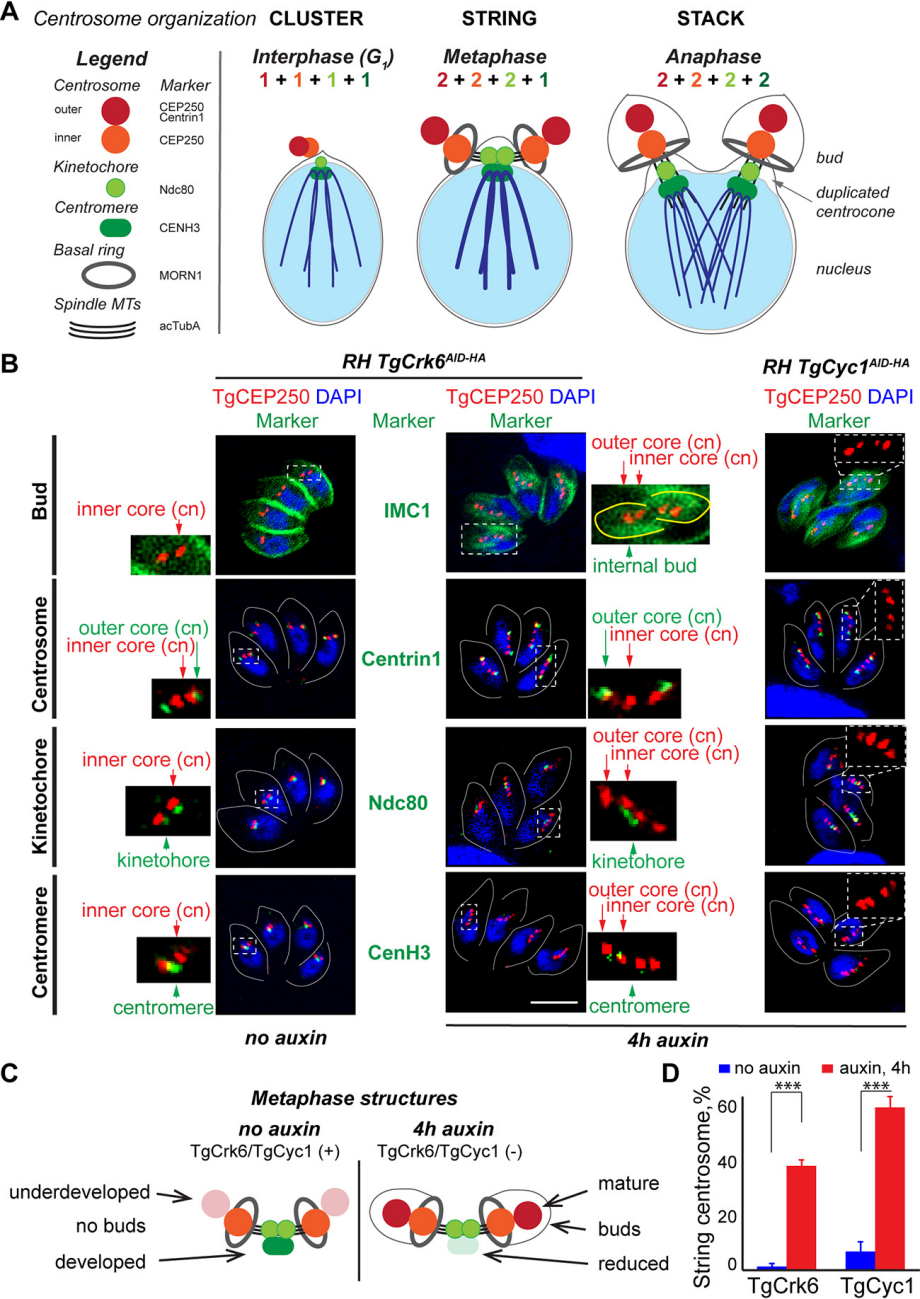
TgCrk6/TgCyc1 complex controls metaphase-to-anaphase transition. (A) Schematics of the interphase, metaphase, and anaphase nucleus and perinuclear structures of the *Toxoplasma* tachyzoite. Two cores of bipartite centrosome expand, duplicate, and separate, forming a distinctive pattern: a cluster in interphase, a string in metaphase, and a stack in anaphase. Present throughout the cell cycle, kinetochore and centromere segregate in anaphase of mitosis, changing the stoichiometry of the mitotic structures (colored numbers). The key mitotic structures and corresponding markers are listed in the legend on the right. (B) IFA analysis of the metaphase in the tachyzoites expressing or lacking TgCrk6 or TgCyc1. To visualize mitotic structures, tachyzoites expressing TgCEP250^Myc^ (α-myc/α-rat IgG Fluor 568, inner and outer core of centrosome marker) were costained with organelle markers using the following antibodies: α-CenH3/α-mouse IgG Fluor 488 (centromere), α-Ndc80/α-mouse IgG Fluor 488 (kinetochore), α-Centrin1/α-mouse IgG Fluor 488 (outer centrosome), α-IMC1/α-mouse IgG Fluor 488 (bud), and DNA stain DAPI. The left panel shows undisturbed metaphase organization (no auxin). Two panels on the right show changes invoked by degradation of TgCrk6 or TgCyc1. Enlarged images on the side demonstrate relative positions of the visualized organelles (red and green channel only). Scale bar, 5 μm. (C) Summary of the IFA analysis in panel B. Schematics show metaphase structures of the parasites expressing (no auxin) and lacking (4 h auxin) TgCrk6/TgCyc1 complex. (D) Quantification of the string centrosome organization in tachyzoites expressing or deficient in TgCrk6 or TgCyc1. Centrosomes were examined in 100 random vacuoles in three independent experiments. The mean and SD values are show on the plots.

Complex mitosis in *Toxoplasma* involves numerous perinuclear and nuclear structures (Fig. 5A) (5–9, 17, 18, 30). Previous studies showed sequential duplication and segregation of the outer and inner cores of centrosome, kinetochores, and centromeres and proposed how these events associate with specific cell cycle phases. While the kinetochores and centromeres simply duplicate, the *Toxoplasma* bipartite centrosome undergoes significant structural changes (7, 9, 18). The outer and inner centrosomal cores cluster in the interphase and form a string or a stack in the metaphase and anaphase of mitosis, respectively (Fig. 5A) (7, 30). To find out whether components of the mitotic structures are affected by TgCrk6 or TgCyc1 deficiency, we examined each constituent of the bipartite centrosome, kinetochore, and centromere in the TgCrk6 and TgCyc1 AID mutants coexpressing endogenously tagged TgCEP250^myc^ that facilitates visualization of both centrosomal cores (Table S1) (7, 9). The structures were examined in the parasites normally expressing (−aux) or deprived of TgCrk6 or TgCyc1 for 4 h (+aux). Detection of the structures using individual markers revealed that stalled parasites largely retained stoichiometry of the metaphase structures (Fig. 5B and C). Duplicated centrosomal cores (TgCEP250 and Centrin1) and kinetochore (TgNdc80) were present in the proper ratios, but we could not detect the centromere marker TgCenH3. This result and the fact that auxin-treated TgCrk6 and TgCyc1 AID parasites were unable to resolve centrocone confirmed the metaphase block induced by the lack of TgCrk6/TgCyc1. This finding also suggests that centromere dysfunction is a reason for metaphase arrest.

While metaphase block caused by TgCrk6/TgCyc1 deficiency did not affect duplication of the centrosomal cores, we noticed significant changes in the core’s maturation (Fig. 5B). Normally, coiled-coil protein TgCEP250 translocates to the outer core in postmetaphase, signifying a core maturation (7, 9). As a result, four TgCEP250-positive centrosomal cores are visible only during anaphase and cytokinesis (7). On the contrary, approximately 40% of metaphase-arrested TgCrk6/TgCyc1-deficient vacuoles contained a mature centrosome displaying four fully developed TgCEP250-positive cores arranged in a string structure (Fig. 5B to D and Table S1). Since the outer core maturation coincides with bud development (cytokinesis), the string centrosomes of TgCrk6- and TgCyc1-depleted tachyzoites were associated with growing internal buds (Fig. 5B, IMC1). Therefore, we concluded that the TgCrk6/TgCyc1 complex predominantly regulates intranuclear events such as chromosome segregation and has little or no effect on the concurrent budding process. The uniform and abundant retention in the metaphase suggests that the checkpoint block is a primary cause of the TgCrk6 and TgCyc1 growth deficiency. The characteristic arrest at the metaphase-to-anaphase transition further suggests that this checkpoint, although regulated by noncanonical *Toxoplasma*-specific complex TgCrk6/TgCyc1, is analogous to the spindle assembly checkpoint (SAC) in other eukaryotes (31).

### *Toxoplasma* SAC is regulated by phosphorylation.

Spindle assembly checkpoint provides fidelity of the genetic inheritance, ensuring a proper attachment of the chromosomes to the spindle fibers during metaphase (32, 33). In studied model eukaryotes, phosphorylation-directed proteolysis coordinates key SAC events (34, 35). Upon checkpoint satisfaction, mitotic CDK1/cyclin B complex activates large ubiquitinating machinery, the anaphase promoting complex or cyclosome (APC/C), that releases the protease separase from its inhibitory complex with securin. Released separase aids segregation of the sister chromatids to the opposite poles of the spindle, followed by packaging into the daughter nuclei. This event marks anaphase and exit from mitosis (36–38). Despite its vital importance, the spindle assembly checkpoint has not been evaluated in apicomplexan parasites. Our new model of the *Toxoplasma* metaphase arrest by conditional manipulation of the TgCrk6 and TgCyc1 levels offers a convenient tool to decode key events of apicomplexan mitosis coupled to budding.

**FIG 6:**
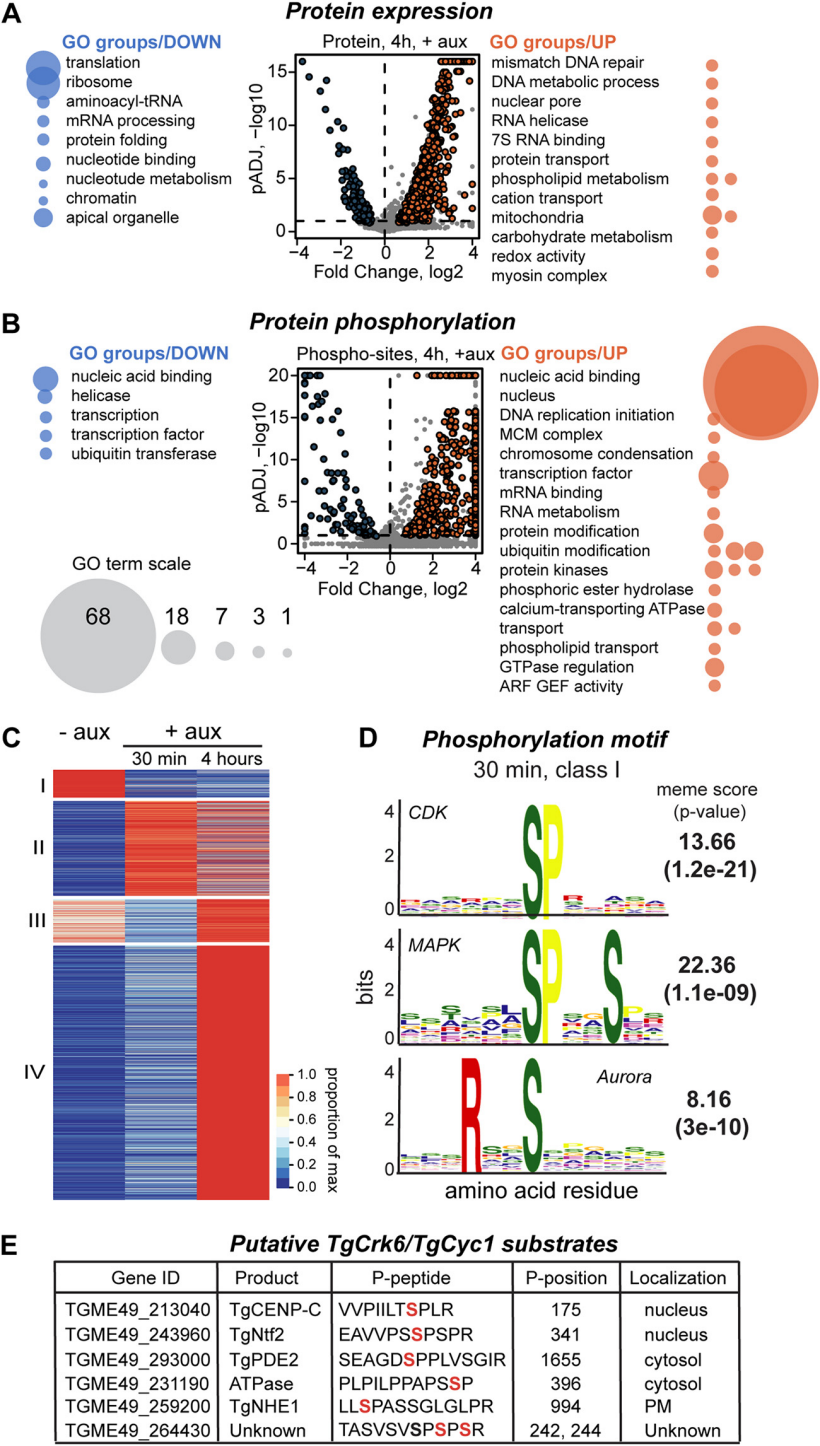
Spindle assembly checkpoint in *Toxoplasma* is regulated by phosphorylation and proteolysis. (A and B) Volcano plots show changes of the protein expression level (upper graph) and of the phosphosite intensity (lower graph) at the checkpoint block induced by TgCyc1 downregulation after 4 h. GO term enrichment analysis was performed on upregulated (orange) and downregulated (blue) groups. Results are shown on the sides of each plot. The bubbles reflect the size of individual pools. (C) The heat map displays global changes of the protein phosphorylation caused by auxin-induced TgCyc1 degradation for 30 min and 4 h. The proteins are organized according to the similarity in phosphorylation by K-means and combined into four classes based on the temporal dynamic of phosphorylation. (D) The phosphorylation motifs affected by 30 min of TgCyc1 degradation were deduced using MoMo software. Three dominant motifs and corresponding scores are shown. The responsible kinase family is indicated on the corresponding plot. (E) Table of the putative TgCrk6 substrates. The list was created based on the reduction of phosphorylation intensity within a proline-driven motif caused by the lack of TgCrk6 and TgCyc1 for 30 min. Affected phosphoserine residue is shown in red.

We performed quantitative proteome analysis of TgCyc1-deficient parasites at the optimal checkpoint block time, 4 h (Fig. S4A and B). As predicted of a SAC arrest, the lack of mitotic cyclin TgCyc1 significantly changed global protein expression and intensity of phosphorylation (Fig. 6A and B and Table S3 and S4; 20 to 50% of global tachyzoite proteome) (34, 35). Comparative analysis of asynchronous (−auxin) and metaphase arrested populations (+auxin, 4 h) showed that more proteins had elevated rather than reduced expression and phosphomodification. Although the proteomes were largely composed of the proteins with unknown function (60 to 95% of a total proteome), to gather information on the *Toxoplasma* metaphase, we ran Gene Ontology (GO) analysis using available annotations (Fig. 6A and B and Table S5). Mitosis takes place in the nucleus. Consequently, our analysis detected dominant changes in the nuclear factors’ phosphorylation accompanied by increased expression of the DNA repair and nucleus maintenance proteins. Phosphorylation changes also affected DNA replication and chromosome condensation machineries. Our data suggest that, during metaphase, degradation represses mRNA translation but phosphorylation controls gene transcription. As expected of the SAC regulation, ubiquitination was a dominant proteolytic mechanism, and it was mainly regulated by phosphorylation (37, 39). Expansion of the nuclear envelope is a large biosynthetic effort in mitosis. Accordingly, we detected elevated production of the factors playing a role in the phospholipid metabolism and altered phosphorylation of the corresponding transport machinery. Previous studies showed drastic remodeling of mitochondria during mitosis, and we now detected corresponding changes at the protein expression level (40). Metaphase block upregulated phosphorylation of several protein kinases, including splicing (DYRK, SPRK, and PRP4), ERK7, and TgTKL2 kinases, whose role in mitosis had not been demonstrated yet. Since mitosis in tachyzoites is coupled to cytokinesis (budding), we found altered expression and phosphorylation of the components of apical organelles as well as protein transport machinery that support assembly of the daughter cytoskeleton. Altogether, our global proteomic analysis confirmed mitotic block regulated by novel TgCrk6/TgCyc1 complex. We found that *Toxoplasma* metaphase arrest is a mixture of canonical and parasite-specific processes. The GO analysis showed repressed protein translation and nucleotide metabolism combined with upregulation of mitotic processes, such as DNA repair, nuclear pore function, controlled proteolysis, and production of nuclear and cellular membranes. A large portion of the proteomes were novel, indicating a bigger role of the parasite-specific processes in metaphase function.

10.1128/mbio.03561-21.4 FIG S4 *Toxoplasma* metaphase is regulated by phosphorylation. (A) Schematics of the tachyzoite cell cycle show population changes due to metaphase arrest. Drawings depict morphological transformations of the dividing tachyzoite. Red wave represents enrichment of the mitotic parasites after 4 h of auxin-induced degradation of TgCrk6 or TgCyc1. Purple, centrosome (marker, Centrin1); green, centrocone and basal complex (marker, MORN1); blue, nucleus (marker, DAPI); black, surface alveoli (marker, IMC1). (B) Summary of the proteomics analysis of TgCrk6-AID and TgCyc1-AID models. (C) The phosphorylation motifs affected by 4 h of TgCyc1 degradation were deduced using MoMo software. Two dominant motifs and corresponding scores are shown. The responsible kinase family is indicated on the corresponding plot. (D) Alignment of the CENP-C domain (blue box) residues of the human CENP-C and putative *Toxoplasma* analog TgCenP-C. Phospho-modified threonine is in red. Conserved residues are indicated with asterisks. Download FIG S4, TIF file, 1.1 MB. Copyright © 2022 Hawkins et al. 2022 Hawkins et al. https://creativecommons.org/licenses/by/4.0/ This content is distributed under the terms of the Creative Commons Attribution 4.0 International license.

10.1128/mbio.03561-21.7 TABLE S3 TgCyc1 quantitative proteome/phosphoproteome data and statistical analysis. Download Table S3, XLSX file, 7.0 MB. Copyright © 2022 Hawkins et al. 2022 Hawkins et al. https://creativecommons.org/licenses/by/4.0/ This content is distributed under the terms of the Creative Commons Attribution 4.0 International license.

10.1128/mbio.03561-21.8 TABLE S4 TgCrk6 quantitative proteome/phosphoproteome data and statistical analysis. Download Table S4, XLSX file, 15.7 MB. Copyright © 2022 Hawkins et al. 2022 Hawkins et al. https://creativecommons.org/licenses/by/4.0/ This content is distributed under the terms of the Creative Commons Attribution 4.0 International license.

10.1128/mbio.03561-21.9 TABLE S5 GO term analysis of the global TgCyc1 proteome/phosphoproteome. Download Table S5, XLSX file, 0.02 MB. Copyright © 2022 Hawkins et al. 2022 Hawkins et al. https://creativecommons.org/licenses/by/4.0/ This content is distributed under the terms of the Creative Commons Attribution 4.0 International license.

To find out what molecular pathways are regulated by the TgCrk6/Cyc1 complex in tachyzoites, we analyzed the phosphorylation status of the proteins immediately after complex inactivation. We compared phosphoproteomes of the RH TgCyc1^AID-HA^ and RH TgCrk6^AID-HA^ parasites grown in the absence (0 min) or presence (30 min and 4 h) of 500 μM auxin (Tables S3 and S4). First, we determined phosphorylation motifs affected by a brief absence of TgCyc1. The meme analysis revealed specific loss or reduced phosphorylation of the peptides carrying CDK, mitogen-activated kinase (MAPK), and Aurora kinase type motifs after 30 min of TgCyc1 deficiency (Fig. 6D). Furthermore, CDK and Aurora kinase motifs remain underphosphorylated during 4 h of TgCyc1 deprivation (Fig. S4) (34, 41, 42). This finding strongly supports our main hypothesis that the TgCrk6/TgCyc1 complex is a central mitotic regulator, because removal of these factors affected the activity of the major mitotic kinases. According to the calculated meme score, TgCrk6 had a strong preference for serine phosphomodification within the xS*Px sequence that matches a short CDK motif identified in other eukaryotes (43). To identify immediate effectors of the TgCrk6/TgCyc1 complex, all the differentially phosphorylated sites were divided into four classes based on the temporal dynamics (Fig. 6C). We first selected peptides for which phosphorylation decreased during 30 min of TgCrk6 and TgCyc1 deficiency and remained low for the duration of the checkpoint arrest (4 h) (class I). We then narrowed down this group to peptides containing a predicted TgCrk6 phosphorylation motif, xS*Px. A total of six peptides (and proteins) satisfied the selection conditions (Fig. 6E).

Among the most prominent TgCrk6/TgCyc1 substrates was the TGME49_ 213040 (TgCenP-C) factor, analogous to centromeric protein CENP-C in other eukaryotes (44). Coincidently, TgCenP-C was also detected in the TgCrk6 pulldown (Fig. 3A), making this protein a strong candidate for TgCrk6/TgCyc1 immediate effector. CENP-C is a component of the inner kinetochore that holds sister chromatids together and mediates a physical link to spindle microtubules during active SAC (44, 45). Thus, the TgCrk6/TgCyc1-controlled TgCenP-C phosphorylation may be one of the central pathways of SAC regulation in *Toxoplasma*. Our screen also identified a nuclear transport factor, TGME49_243960 (TgNtf2). NTF2 proteins control protein transport through the nuclear pore (46). Since the nuclear membrane remains intact during mitosis in *Toxoplasma*, the trafficking across the nuclear membrane becomes of vital importance. It is possible that the TgCrk6/TgCyc1 complex modulates TgNtf2 activity during active SAC. We also detected reduced phosphorylation of unknown protein TGME49_264430, the expression of which is limited to the apicomplexan Sarcocystidae family. More studies are needed to confirm the role of TGME49_264430 in *Toxoplasma* SAC. Taking factor localization into consideration, phosphodiesterase TgPDE2 (TGME49_293000), arsenite-activated ATPase (TGME49_231190), and Na^+^/H^+^ exchanger TgNHE1 (TGME49_259200) are less likely to be true substrates of the TgCrk6/TgCyc1 complex.

## DISCUSSION

Mitosis controls segregation of duplicated chromosome into daughter cells. Elaborate mitotic events are divided into four major subphases: prophase, metaphase, anaphase, and telophase. The key event of mitosis is attachment of duplicated chromosomes to the spindle fibers followed by separation of the sister chromatid at the opposite spindle poles in metaphase and anaphase, respectively (31, 33). In addition, so-called open mitosis organizes dissolution of the nuclear membrane in prophase and nuclear envelope restoration in telophase. Some eukaryotes, such as budding yeast dividing by closed mitosis (nuclear membrane remains intact), incorporate elements of cytokinesis into each mitotic subphase (47). Apicomplexans, including *Toxoplasma*, use a semiclosed mechanism, creating a temporal opening near the spindle compartment centrocone (4, 19). Similar to budding yeast, chromosome segregation in *Toxoplasma* endodyogenic division is coupled to cytokinesis (budding). However, *Toxoplasma* does not encode orthologs of the factors known to coordinate mitosis and budding, suggesting that apicomplexans have evolved alternative approaches to control the same process. Furthermore, apicomplexans lack orthologs of the central cell cycle regulators of model eukaryotes, which is in accord with the renowned observation of the preserved topology of the cell cycle and not the network of regulators (4, 12). This finding prompted us to focus on the similarity of the process rather than the orthology of the regulators when examining the apicomplexan cell cycle. In accord with this notion, we discovered a novel TgCrk6/TgCyc1 complex that, similar to mitotic CDK1/cyclin B complex in mammalian cells, regulates conventional processes of chromosome segregation but within the context of concurrent budding.

Although the TgCrk6/TgCyc1 complex acts as the CDK1/CycB analog, *Toxoplasma* lacks the entire network of Cdk1/CycB complex regulators, implying alternative, parasite-specific pathways to control mitotic progression. Mammalian CDK1/cyclin B complex is regulated by alternating activities of cdc25 phosphatase and Wee1/Myt1 kinases that are not found in apicomplexan genomes (4, 48). It explains why TgCrk6 does not have a tyrosine or a threonine that undergoes inhibitory phosphorylation by Wee1 kinase or activation by cdc25 phosphatase. Nevertheless, TgCrk6/TgCyc1 complex has a precise timing of activity in metaphase that occupies only a fraction of mitosis. The TgCyc1 oscillation is not sufficient to justify the timing because of the early TgCyc1 emergence in late S phase, suggesting additional mechanisms of TgCrk6/TgCyc1 complex control. What are the mechanisms that turn on and off the mitotic TgCrk6/TgCyc1 complex and thereby regulate SAC? An attractive hypothesis is a sequestration of the active component or regulation by targeted localization. Indeed, the TgCrk6/TgCyc1 complex accumulates in the unresolved centrocone with attached MORN rings characteristic of the metaphase, a place where major SAC events occur. Putative targeting components, then, can be among hypothetical proteins with predicted nuclear localization detected in the TgCrk6 complexes.

The spindle assembly checkpoint regulates secure attachment of chromosomes to spindle microtubules in metaphase and licenses separation of the sister chromatids in anaphase. Our study, for the first time, presented evidence of the functional SAC in *Toxoplasma* endodyogeny and, by doing so, complemented preceding morphological studies and reverse genetics examination of several structural and regulatory components (5, 7–9, 12, 15, 17–19). *Toxoplasma* SAC retained a topology of the conventional mitotic checkpoint: it is prominent and regulated by CDK-related, MAPK, and Aurora kinase activities, and the removal of central regulators blocked chromosome segregation. To execute conserved SAC functions, *Toxoplasma* evolved a novel mitotic TgCrk6/TgCyc1 complex that is not entirely conserved among apicomplexans. Expression of the cyclin TgCyc1 is limited to the coccidian branch of apicomplexan parasites. Examination of the TgCrk6/TgCyc1 network revealed a major pathway that parasites employ to control chromosome attachment to spindle fibers during metaphase. Several lines of evidence suggest that the TgCrk6/TgCyc1 complex targets a centromere-associated network (CCAN) that is greatly reduced in apicomplexans. The centromere is a specialized chromosome region responsible for attachment to spindle microtubules. The attachment engages multiple CCAN components assembled in the inner kinetochore that, in turn, recruits the KMN network, connecting centromeres to microtubules (49). We determined that degradation of the *Toxoplasma* TgCrk6/TgCyc1 complex reduced phosphorylation of the putative inner kinetochore protein TgCenP-C. Coincidently, in animal cells CDK1-dependent phosphorylation of the conventional CENP-C is required for proper localization of the factor and stable interaction with core centromere protein CENP-A (TgCenH3 ortholog) (44, 45). Despite their resemblance in the regulatory step, different CENP-C residues are modified in *Toxoplasma* and human cells, S^175^ and T^734^, respectively. Sequence alignment showed that CDK1-regulated T^734^ is not conserved in *Toxoplasma*, possibly due to highly deviated amino acid sequence of putative TgCenP-C, which also includes parasite-specific N-terminal extension containing TgCrk6-regulated S^175^ (Fig. S4D). A difference in CENP-C regulation suggests alternative control of kinetochore assembly in parasites, which, however, most likely leads to an outcome similar to that observed in conventional models (45). The fact that the TgCenH3 marker was lost on the kinetochores of the metaphase-arrested tachyzoites strongly supports a role for TgCenP-C phosphorylation in stabilization of the TgCenH3-TgCenP-C complex and assembly of the functional kinetochore. Coincidently, we detected TgCenH3 and TgCenP-C in the isolated TgCrk6 complexes that further verifies the role for the TgCrk6/TgCyc1 complex in *Toxoplasm*a CCAN function.

In addition to conventional events, the endodyogenic division must complete parasite-specific tasks staged to mitosis (1). One of the major tasks is coordination of chromosome segregation and assembly of internal daughters. However, our data indicate that budding control is not a part of the SAC mechanism. During TgCrk6/TgCyc1-dependent metaphase arrest, internal buds continue to grow, although at a slightly reduced rate, suggesting that regulators other than the SAC complex TgCrk6/TgCyc1 mediate cross talk with the budding machinery. It also implies that the budding is regulated at the initiation point prior to SAC, and once buds start to grow, the process becomes independent of chromosome segregation, which explains often-observed empty zoites in various mitotic mutants (7, 50, 51). Interestingly, analyzing TgCrk6 complexes, we detected a recently reported budding regulator, DNA binding factor TgAP2IX-5 (52, 53). Similar temporal expression and localization of TgCrk6 and TgAP2IX-5 suggests that TgCrk6 interacts with TgAP2IX-5 prior to and independent of TgCyc1. Since TgCrk6 downregulation does not affect the bud initiation, the likely role of TgCrk6 is to inactivate TgAP2IX-5 in mitosis after the budding starts. If, in fact, the TgCrk6/TgAP2IX-5 and TgCrk6/TgCyc1 complexes are mutually exclusive, then it offers a convenient mechanism for temporal control of the budding initiation and chromosome segregation. Alternatively, the other *Toxoplasma* Crks may regulate coupling of the budding and chromosome segregation. The most plausible candidates are cytoplasmic TgCrk4 that is expressed in place of budding and TgCrk5 functioning in the S-phase, where the budding is initiated (12, 54).

A Cdk-related kinase, TgCrk6, is conserved across apicomplexans that use different modes of replication (12, 55). While some coccidian representatives use binary division (single budding cycle), the majority of apicomplexans divide by multinuclear replication that employs two types of mitosis with different goals and rules (2, 3). The goal of unconstrained chromosome segregation during asynchronous nuclear phase is to allow reentry into the S phase. On the contrary, segregation of the chromosomes linked to cytoskeletal components of the future daughter characteristic of the budding stage should conclude with exit from mitosis that requires coordinated assembly of the cytoskeleton and invasion machineries and to prepare for the upcoming G_1_ phase. Therefore, we expect significant changes in the SAC regulation that functions during nuclear or budding phases. Plasmodium falciparum dividing by multinuclear replication, called schizogony, expresses orthologs to TgCrk6 kinase P. falciparum CRK (PfCRK4) that play a role in both DNA replication (S-phase function) and mitosis (55). Although the PfCRK4-deficient parasites arrest with a spindle hemisphere representing a metaphase block, the concurrent deficiency of DNA replication may be a reflection of liberated reentry into the S phase. Unfortunately, the PfCRK4 function has only been demonstrated during the nuclear cycle. Whether PfCRK4 plays a role in the SAC of the synchronized final budding cycle needs to be examined using a different experimental approach with focus on the last stage of replication. Furthermore, Cdk-type kinases tend to work in complexes. Unlike TgCrk6, which forms a complex with TgCyc1 and functions in the budding cycle, a cyclin partner for PfCRK4 has not been found yet, suggesting alternative complexes contribute to SAC regulation during nuclear and budding phases.

In conclusion, we identified central regulators of the key mitotic step in *Toxoplasma*, transition from metaphase to anaphase, occurring in conjunction with parasite budding. Our findings provided further evidence of the conserved topology of the apicomplexan cell cycle, although it is controlled by unique molecular machinery that likely coevolved to adapt to parasite needs. Specifically, we demonstrated that novel TgCrk6/TgCyc1 complex regulates not only the conventional functions of the spindle assembly checkpoint but also concurrent parasite-specific tasks. Our study sends a cue to address several important questions. How is the TgCrk6/TgCyc1 complex activated given the absence of canonical regulators in apicomplexan genomes? How does the SAC of the nuclear phase differ from the SAC of the budding phase in multinuclear division, such as *Plasmodium* schizogony? What pathways constitute the mitotic exit in apicomplexans that involves a preparation for egress and invasion? What is the mechanism that allows the S-phase reentry during nuclear cycles of schizogony?

## MATERIALS AND METHODS

### Parasite cell culture.

Parasites were grown in human foreskin fibroblasts (HFF) as described previously (56). Transgenic and mutant parasite lines are derivatives of the T. gondii RHΔ*Ku80Δhxgprt* *AtTIR1* strain (24). Host and parasite strains were tested mycoplasma free (MP Biomedicals). All parasite lines created in the study are listed in the Table S1 in the supplemental material.

### Phylogenetic analysis.

All protein sequences of the annotated cyclins were downloaded from UniProt (57). The multiple-sequence alignments were generated by the MUSCLE algorithm (58) using the R package msa version 1.4.3. Sequences were first compared for amino acid substitution using the Bayesian information criterion (BIC). The substitution model JTT_DCMut was selected to generate an unrooted tree using neighbor-joining (59). The branch length of the generated tree was optimized by maximum likelihood (60). We used bootstrapping (100 times) to test how well the optimized edges of the tree are supported. The calculated bootstrap support values are shown in the final phylogenetic tree. All the procedures of phylogenetic tree optimization were performed in the R package phangorn version 2.8.0 (61). The final phylogenetic tree was generated using R package ggtree with version 3.2.0 (62).

### Construction of the transgenic strains.

Transgenic strains created in the current study are listed in Table S1.

### (i) pLIC-mAID-3×HA-Hxgprt and pLIC-mAID-3×myc-DHFR-TS vectors.

To create AID conditional expression models of *Toxoplasma* proteins, we built the pLIC-mAID-3×HA-Hxgprt vector. To make pLIC-mAID-3×HA-Hxgprt plasmid, a 3×HA epitope of the pLIC-3×HA-Hxgprt vector was excised with AvrII-NdeI and replaced with complementary AID-3×HA fragment from pTUB1-YFP-mAID-3×HA-DHFR-TS-Hxgprt2 vector (24).

### (ii) pLIC-TgCrk6-mAID-3×HA-Hxgprt, pLIC-TgCyc1-mAID-3×HA-Hxgprt, pLIC-TgCrk6-3×myc-DHFR-TS, and pLIC-TgCEP250-3×myc-DHFR-TS constructs.

DNA fragments encompassing the 3′ end of the gene of interest were amplified by PCR and cloned into the corresponding vectors, digested with PacI endonuclease by the Gibson assembly method. Resulting constructs were linearized and transfected into the RHΔ*Ku80Δhxgprt* *AtTIR1* parent or derivative strains.

### Primers.

Primers used in the current study are listed in Table S1.

### Parasite transfection.

Eight million freshly lysing RH tachyzoites were mixed with 8 μg DNA in 100 μl of the cytomix buffer and electroporated with Amaxa electroporator (Lonza). Transfected parasites were recovered for 24 h prior to drug selection. The drug-resistant polyclonal populations were cloned by limiting dilution to obtain clonal populations. Proper recombination at the locus was confirmed by PCR in 3 individual clones. Tagged protein expression was also verified by Western blot analysis and immunofluorescence microscopy.

### Western blot analysis.

Infected HFF monolayers were lysed directly in the Laemmli loading dye, heated at 95°C for 10 min, and briefly sonicated. Alternatively, total lysates were obtained from filter-purified parasites. After separation on the SDS-PAGE gels, proteins were transferred onto nitrocellulose membrane and probed with monoclonal antibodies against HA (rat 3F10; Roche Applied Sciences), myc epitope (mouse; Cell Signaling Technology), and tubulin A protein (mouse 12G10; kindly provided by Jacek Gaertig, University of Georgia). After incubation with secondary horseradish peroxidase (HRP)-conjugated anti-mouse or anti-rat antibodies, proteins were visualized by enhanced chemiluminescence detection (PerkinElmer).

### Immunofluorescent microscopy analysis.

Confluent HFF cultures on glass coverslips were infected with parasites for the indicated times and under specified growth conditions. Monolayers were fixed, permeabilized, and incubated with antibody as previously described (51). The following primary antibodies were used: rat monoclonal α-HA (clone 3F10; Roche Applied Sciences), rabbit α-Myc (clone 71D10; Cell Signaling Technology), mouse α-Centrin (clone 20H5; Millipore Sigma), mouse α-CenH3 (centromere marker; kindly provided by Boris Striepen, University of Pennsylvania, Philadelphia, PA), mouse α-Ndc80 (outer kinetochore marker; kindly provided by Marc-Jan Gubbels, Boston College, MA), rabbit α-MORN1 (centrocone and basal complex stains; kindly provided by Marc-Jan Gubbels, Boston College, MA), and rabbit and mouse α-IMC1 (parasite shape and internal daughter bud strains; kindly provided by Gary Ward, University of Vermont, VT). All Alexa-conjugated secondary antibodies (Molecular Probes, Life Technologies) were used at a dilution of 1:500. Coverslips were mounted with Aquamount (Thermo Scientific), dried overnight at 4°C, and viewed on a Zeiss Axiovert microscope equipped with a 100× objective using the ApoTome slicer. The collected images were processed first using Zeiss Zen software and then in Adobe Photoshop 2020 using linear adjustment when needed.

### Plaque assay.

Confluent HFF monolayers in a six-well dish format were infected with 100 parasites. Cultures were treated with either a vehicle (ethanol) or 500 μM iodoacetamide (IAA) (auxin) to trigger an AID protein degradation. Plaques developed after 7 days were stained and counted as previously described (12). At least three biological replicates of each assay were performed.

### Proteomic analysis. (i) The immune precipitation sample preparation.

To analyze TgCrk6 complexes, the samples were prepared from 2 × 10^9^ parental RHΔ*Ku80Δhxgprt* *AtTIR1* cells and tachyzoites expressing endogenously tagged TgCrk6^AID-HA^ kinase. Parasites were collected by filtration and centrifugation. Total proteins were extracted by incubation in the phosphate-buffered saline solution with 263 mM NaCl, 0.25% NP-40, and proteinase-phosphatase inhibitor cocktail (Thermo Fisher). TgCrk6^HA^ complexes were isolated on α-HA magnetic beads (MblBio) and compared to the nonspecifically bound proteins from the parental parasites. The efficiency of immune precipitation was verified by Western blotting.

### (ii) Immune-precipitated proteome analysis sample preparation.

Protein samples were processed for mass spectrometry-based proteomic analysis using s-traps (Protifi) (63, 64). Briefly, proteins were reduced with dithiothreitol (DTT), alkylated with IAA, acidified using phosphoric acid, and combined with s-trap loading buffer (90% methanol, 100 mM triethylammonium bicarbonate [TEAB]). Proteins were loaded onto s-traps, washed, and digested with trypsin/Lys-C (1:100, wt/wt, enzyme-protein) overnight at 37°C. Peptides were eluted, dried in a vacuum concentrator, and then resuspended in H_2_O–1% acetonitrile–0.1% formic acid for liquid chromatography tandem mass spectrometry (LC-MS/MS) analysis.

### (iii) The global proteome analysis sample preparation.

RH TgCrk6^AID-HA^ and TgCyc1^AID-HA^ tachyzoites were grown under the indicated conditions and collected by filtration. Samples were solubilized with 5% (wt/vol) SDS in 50 mM TEAB, and protein concentrations were determined using the 660 protein assay (Pierce) supplemented with ionic detergent compatibility reagent (IDCR). Equal amounts of protein (200 μg) were processed for LC-MS/MS using s-traps (Protifi) as described above; however, after peptides were eluted and dried in a vacuum concentrator, the peptides were resuspended in 0.1% trifluoroacetic acid–H_2_O for high-pH reverse-phase fractionation (Pierce). A portion of each fraction was removed for global analysis prior to drying. Individual fractions were enriched for phosphopeptides using TiO_2_ nanopolymer beads (Tymora Analytical). Dry peptide fractions were resuspended in 200 μl loading buffer, added to 50 μl beads, and vortexed for 20 min at room temperature. Beads were added to a fitted pipette tip, centrifuged to remove flowthrough, and washed. Eluted and dried phosphopeptides were resuspended in H_2_O, 1% acetonitrile, 0.1% formic acid for LC-MS/MS analysis.

Peptides were separated using a 75-μm by 50-cm C_18_ reverse-phase high-performance liquid chromatography column (Thermo Scientific) on an Ultimate 3000 ultrahigh-performance liquid chromatography (Thermo Scientific) with a 120-min gradient (2 to 32% acetonitrile with 0.1% formic acid) and analyzed on a hybrid quadrupole-Orbitrap instrument (Q Exactive Plus; Thermo Fisher Scientific). Full MS survey scans were acquired at 70,000 resolution. The top 10 most abundant ions were selected for MS/MS analysis.

### (iv) Raw data processing.

Files were processed in MaxQuant (v 1.6.14.0; www.maxquant.org) and searched against the current UniProt Toxoplasma gondii *Me49* protein sequence database. Search parameters included constant modification of cysteine by carbamidomethylation and the variable modifications methionine oxidation, protein N-terminal acetylation, and phosphorylation of serine, threonine, and tyrosine. Proteins were identified using the filtering criteria of 1% protein and peptide false discovery rate. Protein intensity values were normalized using the MaxQuant LFQ function (65).

Label-free quantitation (LFQ) analysis of the global proteome and phosphoproteome was performed using Perseus (v 1.6.14.0), software developed for the analysis of omics data (66). LFQ intensity values were log_2_ transformed and then filtered to include proteins containing at least 60% valid values (reported LFQ intensities) in at least one experimental group. The missing values in the filtered data set then were replaced using the imputation function in Perseus with default parameters (66). Statistical analyses were carried out using the filtered and imputed protein groups file. Statistically significant changes in protein abundance were determined using Welch’s *t* test *P* values and *z* scores.

### Statistical analysis. (i) Global protein expression and phosphorylation changes.

To determine the relative abundance of the phosphorylated peptides, each phosphorylation intensity value was normalized to the intensity of the corresponding protein expression (global proteome). We then introduced an improved Gaussian model to identify proteins or phosphorylated peptides that were significantly altered during checkpoint block induced by TgCyc1 degradation (4 h with auxin) (67–70). In brief, a Gaussian distribution was implemented to model the log_2_ fold change (log2FC) of normalized protein/peptide intensities. Using a sliding window from low to high of average protein/peptide intensity, we modeled the variance changing of log2FC and selected proteins/peptides within a specific range of intensity. For each replicate, the maximum likelihood estimation (MLE) was used to estimate the parameters in Gaussian distribution from each sliding window after removing entities with log2FC higher than the top 95% or lower than the bottom 5%. The *P* value was calculated as the probability of the fitted Gaussian distribution higher or lower than the observed log2FC value when log2FC was higher or lower than the fitted expectation. The false discovery rate (FDR) was used to adjust the *P* value. We selected significantly changed entities based on the criteria of log2FC > 1 and adjusted *P*  < 0.1, or log2FC < −1 and adjusted *P*  < 0.1, consistently in all the replicates.

### (ii) Heatmap construction.

To capture phosphorylation pattern changes due to TgCyc1 deficiency, we compared pairwise significantly changed phosphorylated peptides detected in parasites that were untreated or auxin treated for 30 min or 4 h. We started with building K-means classification for the 0/30-min pair. Akaike information criterion (AIC) and BIC were used to search optimized category numbers of K-means classification. The normalized intensity of each selected peptide was further divided by the maximum intensity of this peptide from checkpoint block time to 4 h, which is represented as a proportion of the maximum in the heatmap. The enriched GO terms for each group of genes are identified by using a hypergeometric test compared with the whole T. gondii genome with an FDR-adjusted *P* value lower than 0.1.

To generate a heatmap of the temporal gene expression of the putative TgCrk6 interactors, we used the normalized mRNA profile of the tachyzoite transcriptome (28). Top hits/interactors were selected as high-probability entities that were enriched more than 5 times in the TgCrk6 pulldown. Corresponding genes were organized by hierarchical clustering on Euclidean distance. *z* score was utilized to depict relative changes in the gene expression across different cell cycle phases (28).

### (iii) Phosphorylation motif search.

The featured motifs of phosphorylated peptides were analyzed by using the motif-x algorithm Soft MoMo v5.1.1 (http://meme-suite.org/tools/momo) (71). For analysis, we selected 13-mer phosphorylated peptides with phosphomodified amino acid residues positioned between 6 residues upstream and downstream of the phosphorylation site. In cases where flanking residues were missing from the MS-identified peptide, the neighboring sequence was extracted from the predicted protein sequence in the T. gondii genome (ToxoDB). The background or control data sets were selected according to the type of amino acids phosphorylated in the query data. The motif sequence was considered when the minimum number of occurrences was over 25 and the *P* value was <1e^−9^.
