# Supplementary figures and images for: Novel CRK-Cyclin Complex Controls Spindle Assembly Checkpoint in Toxoplasma Endodyogeny
Source: mBio. 2022 Feb 8;13(1):e03561-21. doi: 10.1128/mbio.03561-21 (PMC8822342; doi:10.1128/mbio.03561-21)

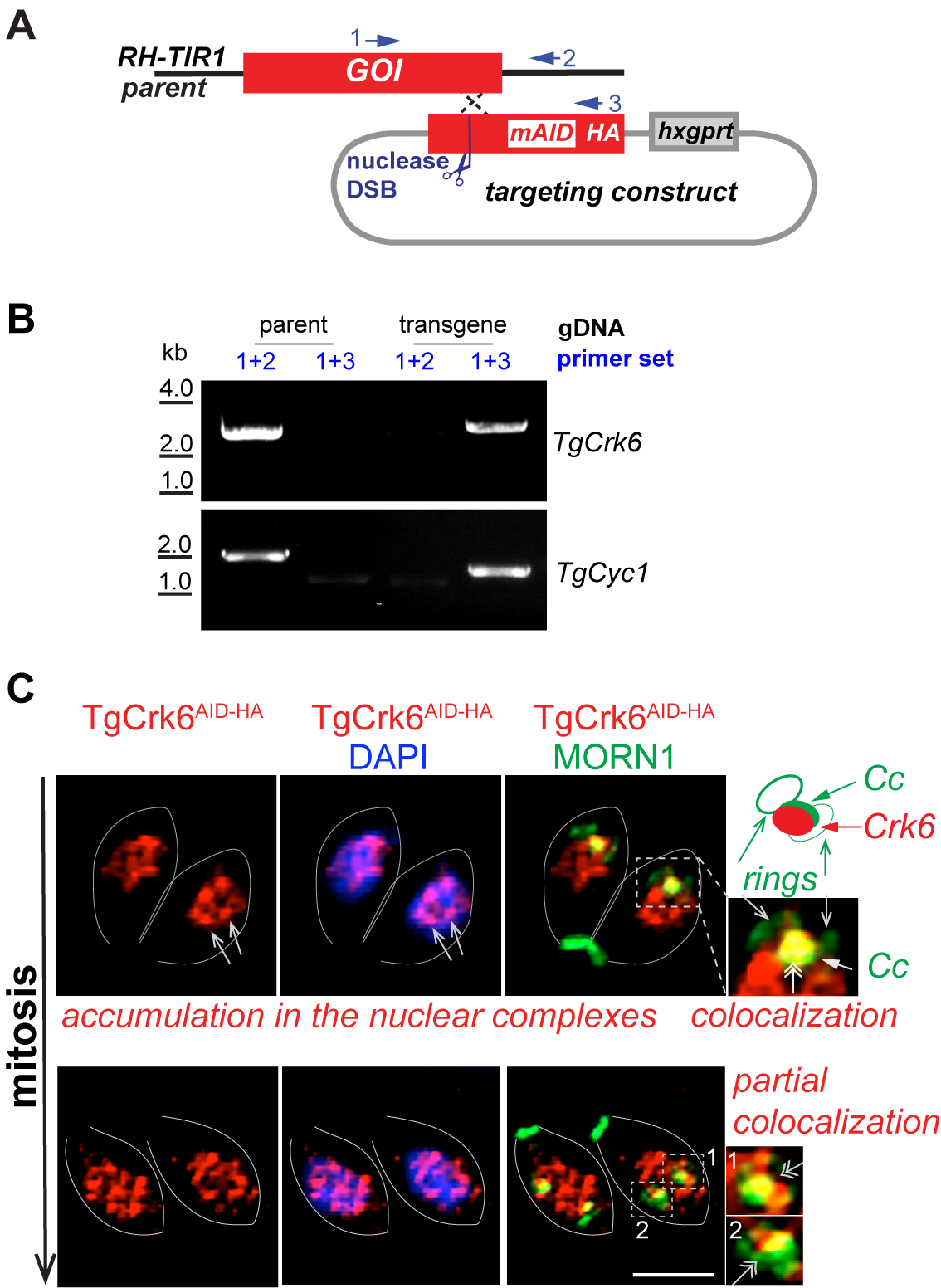

Supplement: FIG S1 [file mbio.03561-21-sf001.tif]

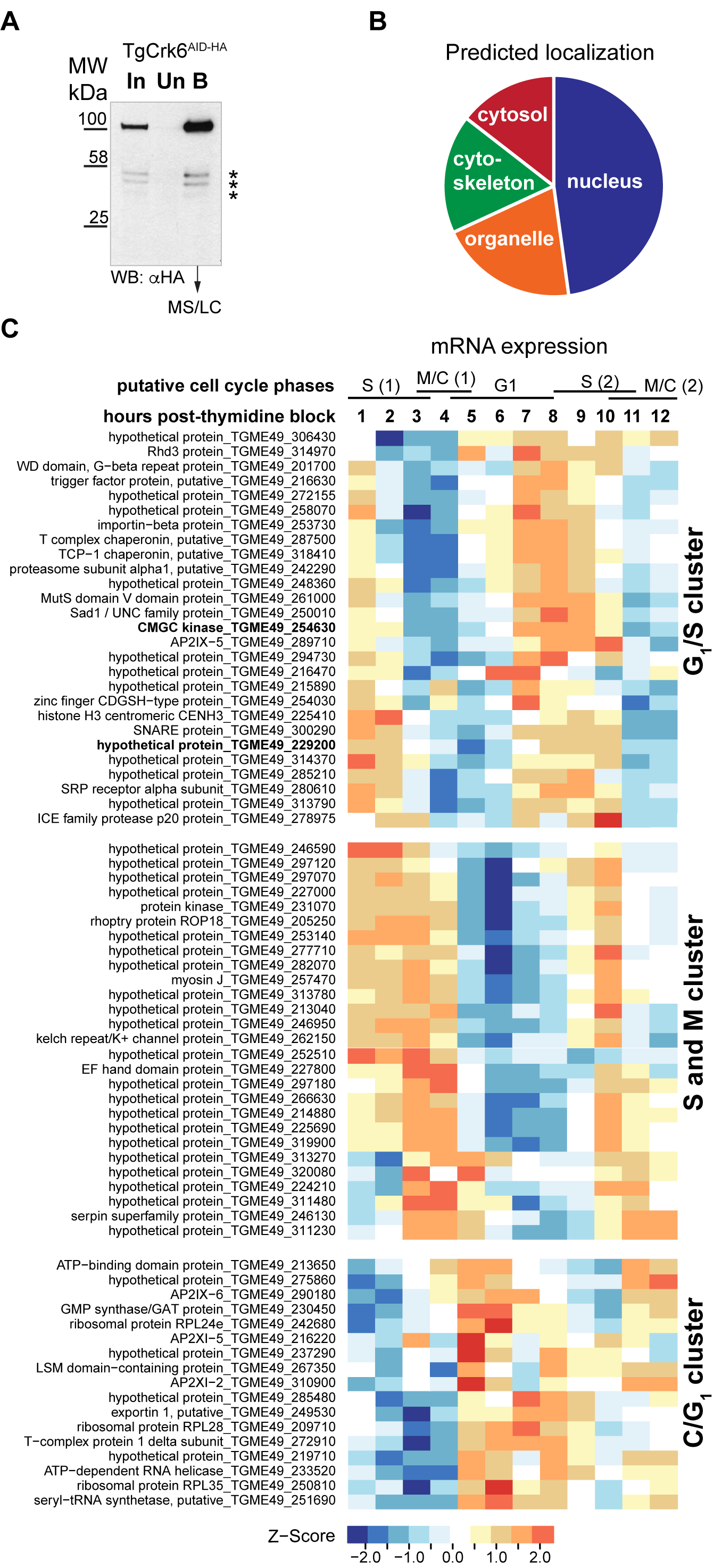

Supplement: FIG S2 [file mbio.03561-21-sf002.tif]

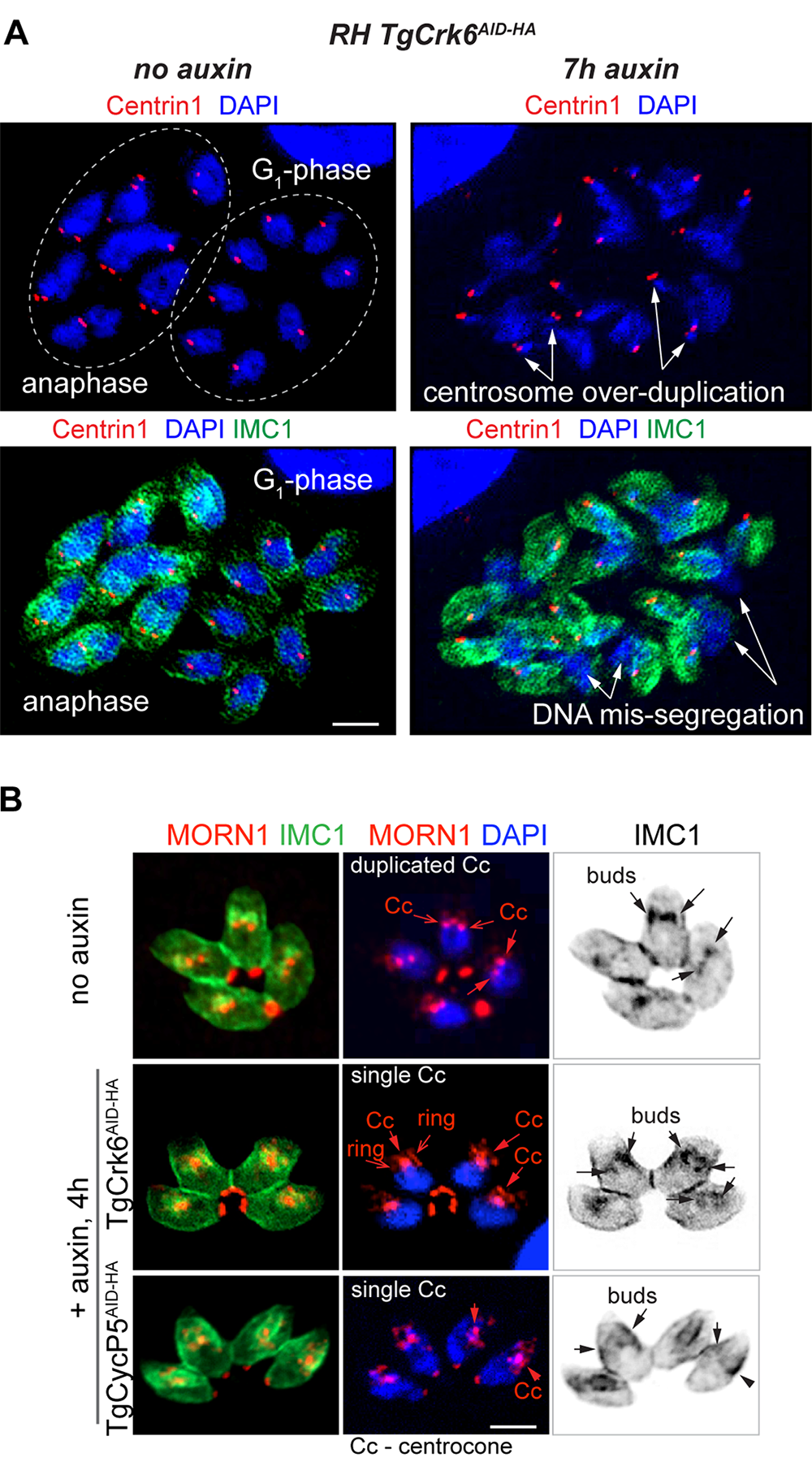

Supplement: FIG S3 [file mbio.03561-21-sf003.tif]

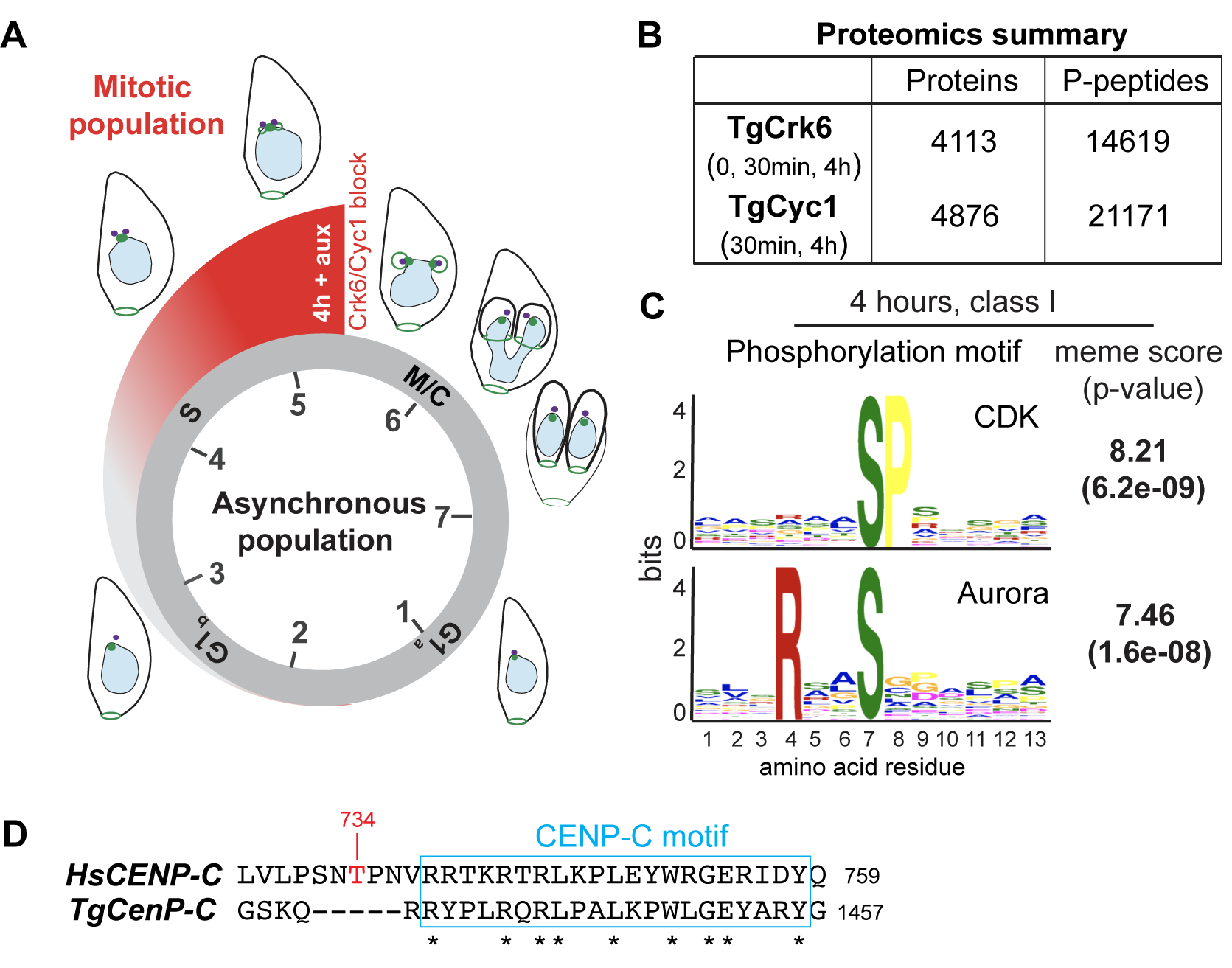

Supplement: FIG S4 [file mbio.03561-21-sf004.tif]
